# Perception of affect in unfamiliar musical chords

**DOI:** 10.1371/journal.pone.0218570

**Published:** 2019-06-21

**Authors:** Eline Adrianne Smit, Andrew J. Milne, Roger T. Dean, Gabrielle Weidemann

**Affiliations:** 1 MARCS Institute for Brain, Behaviour and Development, Western Sydney University, Milperra, NSW, Australia; 2 School of Social Sciences and Psychology, Western Sydney University, Milperra, NSW, Australia; Northeastern University, UNITED STATES

## Abstract

This study investigates the role of *extrinsic* and *intrinsic* predictors in the perception of affect in mostly unfamiliar musical chords from the Bohlen-Pierce microtonal tuning system. Extrinsic predictors are derived, in part, from long-term statistical regularities in music; for example, the prevalence of a chord in a corpus of music that is relevant to a participant. Conversely, intrinsic predictors make no use of long-term statistical regularities in music; for example, psychoacoustic features inherent in the music, such as roughness. Two types of affect were measured for each chord: pleasantness/unpleasantness and happiness/sadness. We modelled the data with a number of novel and well-established intrinsic predictors, namely *roughness*, *harmonicity*, *spectral entropy* and *average pitch height*; and a single extrinsic predictor, *12-TET Dissimilarity*, which was estimated by the chord’s smallest distance to any 12-tone equally tempered chord. *Musical sophistication* was modelled as a potential moderator of the above predictors. Two experiments were conducted, each using slightly different tunings of the Bohlen-Pierce musical system: a just intonation version and an equal-tempered version. It was found that, across both tunings and across both affective responses, all the tested intrinsic features and 12-TET Dissimilarity have consistent influences in the expected direction. These results contrast with much current music perception research, which tends to assume the dominance of extrinsic over intrinsic predictors. This study highlights the importance of both intrinsic characteristics of the acoustic signal itself, as well as extrinsic factors, such as 12-TET Dissimilarity, on perception of affect in music.

## Introduction

### Perception of affect in unfamiliar musical chords

While it is well recognised that complex musical compositions can induce strong emotional responses, it has also been found that even simple musical events such as two- or three-tone combinations (i.e. dyads or triads) can convey affect [1–2]. In this article, we examine affective responses–pleasantness and happiness–to unfamiliar microtonal triads and we identify a number of features of these chords that are associated with these affective responses. Dyads or triads are often tested according to their perceived consonance (e.g. [3–5]) or their perceived happiness [6–8]. In order to explain the origin of these affective responses, studies often focus on whether they are caused by *intrinsic* features such as roughness and harmonicity, or *extrinsic* features such as familiarity and cultural cues (e.g. [9–11]). Extrinsic features are, in part, derived from long-term statistical regularities in music or in co-occurrences of music and non-musical events. It follows from this definition that extrinsic features depend not just on the stimulus itself but also on features not in the stimulus. For instance, the prevalence of a specific chord type (e.g., major, minor, diminished, augmented) in a corpus of music is an extrinsic feature that can be used to model chord-type familiarity and the positive emotions that are typically associated with familiar experiences. Similarly, the prevalence of co-occurrences between chord types and happy or sad events (e.g., in movies [12]) is an extrinsic feature that can be used to model long-term associative learning. On the other hand, intrinsic features do not make use of long-term statistical regularities. For example, psychoacoustic features such as the roughness or harmonicity of a chord are not calculated from long-term statistical regularities, hence they are intrinsic [13].

The above-defined distinction between intrinsic and extrinsic features is useful for the following reasons. If an intrinsic feature is demonstrated to be typically involved in specific affective responses, this same feature–because it does not rely on long-term statistical regularities–has the capacity to induce similar affects across different musical cultures and so is a potential musical universal. By definition, this capacity cannot exist for extrinsic features because they are derived, in part, from a statistical analysis/exposure of/to a musical culture. The potential causal antecedence of intrinsic features makes them particularly interesting, as identifying them gives us insight into why music takes the forms it does and why it communicates in the way it does [14]. They are also interesting to a composer who wishes to create new forms or to use musical features that have the capacity for a broader cultural reach.

Music perception studies looking at the role of extrinsic and intrinsic predictors by observation of the profound differences in musical preferences across time and culture (e.g. [15–16]) have often tested only a limited number of intrinsic features (e.g. [17–18]). Additionally, often only Western musical stimuli are used with Western enculturated participants, who have had regular and longitudinal exposure to Western music. This makes it difficult to test for the effects of intrinsic predictors, as participants are very familiar with the statistical regularities for this particular musical culture. In order to test which and to what extent intrinsic features are having a consistent effect on affective responses, familiarity and prior exposure to stimuli should be minimized as much as possible. This can be done by either finding participants naïve to Western music, or by using unfamiliar music with statistical regularities unknown to the participants. Achieving the first is difficult, as demographic groups naïve or limited in their exposure to Western music are becoming increasingly rare. Besides, studying such populations requires costly and time-consuming fieldwork. An example of such a cross-cultural study was done by McDermott et al. [19] who tested the effect of several intrinsic qualities of chords on consonance and dissonance perception in an Amazonian population. Their results showed support for the hypothesis that preference for consonance over dissonance can be absent in cultures that have not had regular exposure to Western music. However, such studies would be difficult to replicate and as such to establish the reliability of the findings. Occasionally, studies use music affiliated with non-Western cultures to examine affective responses to music (e.g. [9]). However, these systems often have similarities with Western systems, such as the use of octaves. Also, due to a growing multicultural society and the easy spread of music through the internet, one can have access and be exposed to multiple musical cultural systems, which is hard to control for.

The aim of this research is to apply three methodological innovations in an alternative and systematic approach to examine affective responses to unfamiliar musical chords. First, microtonal systems have been proven to be particularly helpful in discovering underlying innate mechanisms of music perception outside of a specific cultural context [20–24, 14]. Second, non-microtonal studies have often considered only a rather limited number of distinct features (such as only roughness and/or harmonicity). This paper combines multiple novel and established extrinsic and intrinsic predictors to model affective responses in a systematic approach. Third, both pleasantness and happiness responses will be tested with the same stimuli and participants allowing them to be compared.

To this end, this study assessed in two experiments the affective evaluation of unfamiliar musical chords outside the familiar Western musical context, using triads from the Bohlen-Pierce (BP) system, in order to examine the impact of intrinsic features, as well as the effect of each chord’s dissimilarity to the culturally prevalent 12-tone equal temperament (12-TET) tuning system. The Bohlen-Pierce system does not use the octave as a repetition interval and it contains many intervals that are distinct to those found in conventional Western music. Affective evaluation was measured as ratings of consonance (pleasant/unpleasant) and valence (happy/sad). To ensure that differences in chord ratings were not due to a general misconception of the term consonance by non-musicians, we measured ratings of perceived pleasantness and the term consonance was not mentioned to participants. Participants were clearly instructed to indicate what the chord expressed (perceived emotion), rather than how the chord made them feel (felt emotion).

The intrinsic features used in this study are mathematically modelled and are a combination of novel and well-established predictors, namely roughness, harmonicity, spectral entropy and average pitch height. We modelled one single extrinsic feature, 12-TET Dissimilarity, which is estimated as the sum of differences between the tones of each BP chord and the tones in the nearest twelve-tone equal temperament (12-TET) chord. Additionally, the musical sophistication of each participant was measured to establish whether it moderated the impact of any of the intrinsic or extrinsic features. This set of intrinsic and extrinsic features provides the basis for a systematic approach in a correlative study of music and affect by using a musical system that has been understudied in music perception research.

### The Bohlen-Pierce system

The Bohlen-Pierce system was independently conceived by Heinz Bohlen, Kees van Prooijen and John Robinson Pierce in the 1970s and 1980s [25]. Bohlen-Pierce (BP) is a tuning system whose intervals contain approximations only of odd integer frequency ratios, instead of a combination of odd and even as found in the Western diatonic-chromatic system. The interval of repetition is a *tritave* of 3:1, which is analogous to the octave of 2:1 in the aforementioned Western systems (there are no 2:1-like intervals in BP). The tritave is typically divided into 13 steps and has many intervals that are close to low-integer ratios. Intervals considered consonant in Western music often closely approximate low-integer ratios (and the converse). In the BP system, it is typically asserted, from a theoretical point of view, that a similar principle will hold. A more detailed description of the scale and its chords can be found in Mathews et al. [26].

From the chromatic BP scale, 78 unique triads can be formed when including the tritave. This number decreases to 66 triads when the *tritave* is excluded. These 66 chords form the stimulus corpus for the current experiment. For experiment 1, the just intonation version of the scale was used, as described by Bohlen [27] in his original paper. Due to slight tuning differences in just intonation, the number of chords tested raises therefore from 66 to 414. The second experiment uses the equal-tempered version of the scale similar to Mathews et al. [26] and tested the 66 chords. Just intonation is a pure tuning using intervals that are based on small integer ratios, which makes the system sound very consonant. These pure intervals do not work for every key, which makes it impractical for some instruments. In equal temperament, intervals do not consist of whole number ratios but are changed slightly. In this way, an octave is divided into twelve equally-sized intervals, making it a very practical tuning that can be used by most instruments.

The cumulative cents positions per “semitone” (i.e. smallest) step used for both experiments are presented in Table 1. The starting tone is represented as 0 cents.

**Table 1:** Cumulative cents positions of the equal-tempered and the just intonation Bohlen-Pierce scales.

Semitone step	Interval name	Equal temperament (cents)	Just intonation (cents)
1	BP first	146.3	133.2
2	BP second	292.6	301.8
3	BP third	438.9	435.1
4	BP fourth	585.2	582.5
5	BP fifth	731.5	736.9
6	BP sixth	877.8	884.4
7	BP seventh	1024.1	1017.6
8	BP eighth	1170.4	1165.0
9	BP ninth	1316.7	1319.4
10	BP tenth	1463.0	1466.9
11	BP eleventh	1609.3	1600.1
12	BP twelfth	1755.6	1768.7
13	Tritave	1901.9	1901.9

From a musical perspective, equally tempered scales are often considered advantageous because of their practicality. As the scale is divided into a certain number of equal steps, it is possible to freely transpose up and down and move between keys, which is advantageous for composers and performers. An argument for using just intonation is that the intervals more precisely correspond to the previously mentioned low-integer ratios, which are theoretically highly consonant. In the case of the Bohlen-Pierce system, the deviation in cents between steps of the equal-tempered and the just intonation BP scale is generally smaller than the deviation between the equal-tempered and just intonation Western equivalents [25] and it would therefore not be surprising if there is not much difference in perception of the two tuning systems. However, the just intonation BP scale was originally described by Bohlen [27] whereas the equal tempered version is most often used for compositions and/or perceptual experiments. In this study, both versions of the scale are experimentally tested.

An interesting characteristic of BP is that some of its triadic structures are analogous to those of Western systems, for example the formation of major chords. The BP major chord (0 6 10 in BP semitone steps) is derived from the harmonic series of a pitch, similar to how a Western major diatonic chord is formed. As the pitches of a major chord approximate quite well integer multiples of the fundamental frequency, the chord is expected to be one of the most consonant chords in the scale.

Only a limited number of studies have assessed consonance of BP chords. Mathews et al. [26] examined the perceived consonance of the 78 possible triads that can be formed within the range of 1 *tritave* of the chromatic equal-tempered version of the BP scale. Participants rated dissonance on a 7-point Likert scale with 7 being “very consonant” and 1 “very dissonant”. The concept of consonance was not specifically explained to the non-musicians. Results from their first experiment showed a large difference in ratings between the chords that were perceived as most consonant and those perceived as most dissonant (see Table 2). A strong Pearson correlation (*r* = .68) was found between the mean dissonance of each of chord as rated by the musicians and non-musicians. Notably, the BP major chord (0 6 10) was not rated as one of the most consonant chords for the musician group, and only the 8^th^ highest rated for the non-musician group.

**Table 2:** Consonance ratings for the most consonant and dissonant rated BP chords in equal temperament (Mathews et al., 1988). Means were taken over 72 responses per chord.

Most consonant				Most dissonant			
Musicians		Untrained		Musicians		Untrained	
Chord	Mean	Chord	Mean	Chord	Mean	Chord	Mean
0,11,13	5.31	0,7,10	4.97	0,1,2	1.61	0,1,2	2.47
0,2,5	5.25	0,2,11	4.494	0,11,12	1.67	0,1,3	2.64
0,5,11	5.22	0,7,13	4.89	0,12,13	1.89	0,12,3	2.75
0,6,8	5.17	0,6,13	4.83	0,9,10	1.89	0,2,3	2.81
0,2,8	5.14	0,8,11	4.81	0,10,11	1.94	0,9,10	3.06
0,3,5	5.03	0,4,7	4.78	0,8,9	2.00	0,4,5	3.06
0,3,13	5.11	0,7,9	4.78	0,1,9	2.06	0,8,9	3.11
0,7,11	5.08	0,6,10	4.75	0,1,13	2.22	0,11,12	3.11

Mathews et al. [22] suggested that the high average dissonance ratings were explained by the presence of one-step intervals, but other possible intrinsic predictors were not further investigated. Interestingly, it appears that there was an influence of musical experience in the musician group. Out of the eight chords rated as most consonant, the first five contain intervals that have a close relation to diatonic intervals. Familiarity with diatonic intervals appeared to affect the ratings of those BP chords. The current experiment is based on that initial study by Mathews et al. [26] but intends to expand on their results by, first of all, testing both the just intonation and the equal-tempered scale in the same experimental paradigm. Second, apart from consonance, valence will be tested in this study as well. Third, by also testing multiple intrinsic predictors and one extrinsic predictor, it is hoped that a more systematic explanation of affective responses will be provided. This is mostly missing in Mathews et al. [26], as they only mention musical sophistication and the presence of one-step intervals as possible explanations for affective ratings.

In a recent study, Friedman et al. [28] used isochronous and semi-random tone sequences from the BP system to test the theory that average pitch height is correlated with happiness/sadness. Similar to the aim of the current experiment, by using tone sequences from an unfamiliar musical system, the effect of average pitch height on perceived valence could be assessed independently from previous exposure to a well-known musical system. As well as finding an association between pitch height and valence, Friedman et al. [28] also tested whether average pitch height had a stronger effect than average interval size, as melodies containing larger intervals have been found to be associated with happy music and melodies containing small intervals with sad music. In their experiment, 114 participants were asked to rate happiness/sadness of a set of the BP tone sequences in two different conditions (Congruent Pitch Height/Interval Size and Incongruent Pitch Height/Interval Size). A main effect for pitch height was found, showing that melodies with higher average pitch were rated slightly happier than the average. No effect of average interval size was found, which might imply that the tuning differences in just intonation and equal temperament in the current study will be negligible as well. Their results partially support prior theories [29–31] that higher average pitch height correlates with higher perceived ratings of happiness.

Even though the stimuli used in this experiment will be unfamiliar to the participants, at the end of the experiment they will have received short-term exposure to the Bohlen-Pierce system and might have gained some familiarity with the system [32]. Statistical learning might therefore occur leading to rapidly formed preferences within the unfamiliar musical system. Support for such rapid acquisition of preferences within BP has been shown by Loui [33]. This newly acquired exposure to the system might have an effect on their affective ratings [33–34]. This short-term learning is not the focus of our study, but an exploratory regression (detailed in the Results section) was conducted in order to estimate the effects of exposure throughout the experiment.

### Intrinsic predictors

As mentioned in the introduction, affective responses to music, such as pleasantness or happiness, can be modelled by intrinsic psychoacoustic predictors. These features suggest which aspects of the acoustic signal might correlate with affective responses. Crucially, such intrinsic features would not depend on prior knowledge of statistical features in the music under examination, hence might have the capacity to be independent of a listener’s musical culture. As detailed below, this study uses several intrinsic features, some of which are novel (or calculated in a novel way) and some of which have previously been used as predictors of consonance and valence in Western tonal systems. It is expected that multiple independent predictors–intrinsic and extrinsic–are simultaneously at play, rather than just one.

#### Roughness

*Roughness* is a well-known phenomenon, first proposed by Helmholtz [35] as an explanation for why some musical intervals are considered dissonant or unpleasant. Roughness occurs when frequency components in the acoustic signal differ only slightly, causing interference within the same critical bandwidth of the cochlear and hence resulting in audible beating (e.g. [3,1,36]). Helmholtz [35] suggested that roughness is an explanation for the experience of dissonance, which has been the most prominent view during the 20th century [37]. This theory of roughness has been challenged by McLachlan et al. [38]. In two experiments, it was found that an increasing number of harmonics in a chord does not increase perception of dissonance, and that perception of dissonance in chords can be altered by training. Their findings, which are highly suggestive of a familiarity and music training effect on dissonance, led to a new model of dissonance based on familiarity and musical experience. The dual-process theory of dissonance proposed by Johnson-Laird et al. [39] assumes a combination of ‘sensory’ and ‘tonal’ dissonance, in which the first is caused by roughness and the latter by top-down cognitive processes, i.e. learned processes of tonality. This theory is based on Western tonal music, but the authors claim it should hold for unfamiliar tuning systems as well.

In the present study, all else being equal, negative correlations of roughness with both pleasantness and happiness ratings are hypothesized. Roughness was calculated for harmonic complex tones with 64 harmonics of relative magnitude 1*/n*, where *n* is the harmonic number. This spectrum provides a reasonable approximation of the spectrum of the piano sound used in the experiment. As the purpose of this study is whether roughness can contribute to perception in a general sense, it is not measured on the acoustic signal itself, but calculated on the theoretical properties of the generated sound.

#### Harmonicity

*Harmonicity* is generally described as the degree of similarity between the spectrum of a tone and a template harmonic complex tone, which consists of numerous frequencies all at integer multiples of a single fundamental frequency [40,24,41]. There are a variety of precise mathematical quantifications of harmonicity (see [42] for an overview of harmonicity models). Harmonicity is thought to contribute to pleasantness independently from roughness–consonance may therefore be not due only to a lack of unpleasant beating, but also to high harmonicity [43]. In this paper, we use a completely novel mathematical quantification of harmonicity, as outlined below.

Similar to roughness, the study’s interest in the contribution of harmonicity to perception in a general way does not require it to be measured from the acoustic signal itself. The harmonicity of a triad is here quantified by the value–at the appropriate location–of the nonperiodic relative triad expectation tensor (a matrix) [44] of a single harmonic complex tone with harmonics taking amplitudes of 1/*n*, where *n* is the harmonic number. The expectation tensors approximate the actual acoustic signal, but do not consider the additional difficulties of pitch perception such as frequency and amplitude masking [45].

The value of the expectation tensor provides a “count” of the number of times a given triad occurs within the harmonic series but, crucially, this count is weighted by the numbers of the harmonics involved in the chord (chords comprising high-numbered harmonics have a lower weight than lower-numbered harmonics); furthermore, perceptual inaccuracies of pitch perception are explicitly modelled to allow for a chord that is similar–but not identical–to one found in the harmonic series to gain an associated count that is penalized by distance. Unpacking these concepts is best achieved by working through a simple example.

Let us consider a chord with four musical pitches (in cents) arranged in a vector in ascending order (0, 400, 700, 1100); hence this is a 12-TET major seventh chord (e.g., C-E-G-B). For example, we also have an associated vector of weights (1, 0.5, 0.5, 1) for those musical pitches. Now imagine the expectation matrix as a discrete two-dimensional graph (a surface) where the horizontal and vertical axes are cents above any given *reference pitch*. We use the following procedure to define each value in this matrix (height of each point of the surface). Let us first choose the first pitch in the above pitch vector as the reference. With respect to that reference, there are three ordered pairs of intervals that define a triad of pitches: (400, 700), (400, 1100), (700, 1100). Each of these pairs has an associated weight which is the product of the weights of their entries; respectively: 0.25, 0.5, and 0.5. So, in the graph at entry (400, 700), we place a value of 0.25; at (400, 1100), we place a value of 0.5; at (700, 1100), we place a value of 0.5; and so on. Now we take the next pitch in the set as the reference, so we start from 400 to get the ordered pair (700–400, 1100–400) = (300, 700). This has a weight of 0.25. So, in the graph at entry (300, 700), we place a value of 0.25.

If the same ordered pair appears more than once in the original pitch vector, the weights are simply added in the expectation matrix. The values in the expectation matrix can, therefore, be thought of as weighted counts of triads in the original vector of pitches. Next, we convolve these “spikes” with a two-dimensional Gaussian kernel to smooth them as a model for inaccuracies or uncertainties of pitch perception. To allow us to calculate harmonicity, we use as our initial pitch vector a large number of harmonics (in these calculations, harmonics 1 to 64, in cents) and weight each harmonic by 1/*n*, where *n* is the harmonic number. From this we construct the expectation tensor, as above. This gives us a “weighted count” of triads within the harmonic spectrum, smoothed to take account of perceptual uncertainty. From this matrix, we can read off the harmonicity of any triad up to one cent resolution. If a given location in the graph (i.e., a given triad) has a high value this means that it occurs frequently in the harmonic series and/or it occurs between low-numbered (hence highly weighted) harmonics.

We therefore anticipate that, all else being equal, harmonicity will be positively correlated with both pleasantness and happiness.

#### Spectral entropy

*Spectral entropy* is a measure conventionally used to quantify the amount of noise in a signal [45–47]. Entropy is usually defined as a measure of disorder or chaos in which a high entropy represents a greater level of disorder and has applications in different fields, such as thermodynamics, information theory and statistics. In digital signal processing, spectral entropy is traditionally calculated from the power spectrum of a signal (although the magnitude spectrum can also be used). This spectrum is then scaled so that its sum is one, hence turning it into a probability mass function, here denoted *x*. The entropy, *H*(*x*), of this probability mass function is then calculated accordingly.

$$H(x)=-\sum_{n=0}^{N-1}x_{n}\log x_{n}$$

Spectral entropy is often used as a timbral descriptor of audio signals (e.g. [48]) but was first suggested as an intrinsic measure for modelling consonance or the “overall tonal affinity of a single pitch class set” in Milne et al. [49]. In the spectral entropy calculation used in this article, the complete sound spectrum of all tones in a pitch set, such as a BP chord, is smoothed with a Gaussian kernel to simulate inaccuracies of pitch perception. The resulting smoothed spectrum is then scaled, as described above, resulting in a probability mass function based on magnitudes that, through the smoothing, also incorporates uncertainty of pitch perception. The entropy of this distribution is then determined.

Spectral entropy, calculated like this, is a way of aggregating the spectral pitch (class) similarities [24,50,14,42,44] all pairs of sounds in a pitch (class) set, such as a chord or scale. This is because the greater the degree of overlap of the partials in two different sounds, the higher the spectral pitch similarity of the two sounds and the lower the overall entropy of the resulting spectrum. While spectral pitch similarity can be applied only to exactly two different spectra, spectral entropy is applied to a single spectrum–the spectrum of the composite sound. To date, spectral entropy has been used to predict the tonal affinity of a variety of microtonal scales [49] but, until this study, no experimental testing has been undertaken to validate these predictions. Similar to roughness, the spectrum of each tone was idealized as a harmonic complex tone with 64 harmonics with magnitudes 1*/n*.

In short, spectral entropy is a measure of the unpredictability or disorder of the spectrum produced by the chord and is expected to have a negative effect on ratings of consonance and valence. In other words, the higher the spectral entropy, the more unpredictable the chord, which is expected to result in a lower rating.

#### Average pitch height

In this study, average pitch height is defined as the mean of the musical pitches in a given chord. So, if a chord *x* has musical pitches (60, 68.78, 74.63 in MIDI pitch) (this is a BP “major” chord whose root is at middle C), its average pitch height is 67.8. Average Pitch Height square will also be included in the models to allow for possible linear, “U”, or inverted “U”-shaped effects of pitch height. This is desirable because we are interested in assessing the effects on ratings of the different chord types independently from their overall pitch. A few experimental studies have tested the effects of average pitch height on perception of happiness/sadness and their results consistently show a positive relationship between pitch height and valence; where higher pitch accords with happy and lower with sad [28,51–52,31]. The association between preference and pitch height is not as robust in the general music perception literature. Several studies have found inverse relationships or an *inverted-U- shaped* function between preference and pitch height (see [53] for an overview of those studies), but it is important to note that the range of frequencies used is often larger in the studies reporting an inverse relationship or an inverted-U-shape. In the previously discussed study by Friedman et al. [28], the positive relation between average pitch and happiness ratings was found despite the unconventional nature of the scale and was independent of interval sizes, which is suggestive of it being a robust effect.

### Extrinsic predictor

#### 12-TET Dissimilarity

Apart from intrinsic characteristics of the sounds actually being listened to, extrinsic factors such as familiarity with a musical system play a part in music perception. As mentioned earlier, these factors are derived from long-term statistical regularities in musical events, but are not necessarily independent of stimulus features. Familiarity plays a significant role in the determination of consonance and dissonance of chords, evidenced by a decrease in dissonance perception with musical experience [54,43,55]. Some intervals in the BP system are quite close to Western intervals, which might positively influence ratings of their consonance and valence [26]. We assume that 12-TET (12-tone equal temperament) is the tuning system participants will be most familiar with, as this and very similar tunings are ubiquitous in Western music. As familiarity cannot be objectively and directly measured, a predictor measuring the dissimilarity between BP chords and Western 12-test chords was included which we named *12-TET Dissimilarity*. Dissimilarity for a given BP chord is therefore calculated as the sum of differences, in semitones (in MIDI pitch) between it and the chord comprising only 12-TET intervals that minimizes this sum of differences. For example, given the BP chord (60, 68.78, 74.63), the 12-TET chord (59.78, 68.78, 74.78) minimizes the sum of pitch distances, hence the former chord’s *12-TET Dissimilarity* is |60−59.78|+|68.78−68.78|+|74.63−74.78| = 0.37. A negative effect is hypothesized for 12-TET Dissimilarity, as lower pleasantness and happiness ratings are expected for chords containing intervals that are more dissimilar from 12-TET intervals.

### Moderator

#### Musical sophistication

*Musical sophistication*, as defined by Müllensiefen et al. [56] is “a psychometric construct that can refer to musical skills, expertise, achievements and related behaviours across a range of facets that are measured on different subscales” (p. 2). This definition focuses on the multi-dimensionality of musical behaviour and includes listening skills, music-production abilities and responses to musical situations. High levels of musical sophistication are found for individuals who show more prevalent and varied musical behaviours which are executed with greater ease [56].

Musical sophistication is expected to moderate the effects of the described intrinsic and extrinsic predictors. Music perception studies often have a clear separation between two participant groups: musicians and non-musicians. However, it is difficult to pinpoint what exactly defines a musician as there is no clear consensus on the requirements of a musician [57]. This experiment will not separate the analysis between two groups (musicians and non-musicians) but rather employ musical sophistication as a continuum and it will therefore be treated as a continuous covariate. Musical sophistication is calculated through the psychometric tool called the Goldsmiths Musical Sophistication Index (GMSI) developed by Müllensiefen et al. [56]. We center the resulting musical sophistication index with respect to the population average of 81.58 and subsequently standardized. The number 81.58 originates from the initial study 147,633 of participants [54]. Musical sophistication is expected to modify the other effects.

To quickly summarize, roughness, spectral entropy and 12-TET Dissimilarity are hypothesized to have a negative effect on affective ratings, whereas harmonicity and average pitch height are expected to positively affect ratings. It is expected that musical sophistication moderates the other effects. Besides, it is expected that the slight tuning differences between just intonation and equal temperament will not have an impact on the ratings of the chords.

### Experiments

In two experiments, the influence of intrinsic and extrinsic features on the perception of consonance and valence of BP chords was tested. Based on previous results by Mathews et al. [26], it is expected that ratings of consonance and valence will be distinctly different for several chords. It is also expected that ratings of chords will not distinctively differ between just intonation and equal temperament.

## Methods

### Experiment 1

#### Participants

Sixty participants took part in the study (mean age = 25.7 years, SD = 10.55, 45 females). Participants included 46 undergraduate psychology students from Western Sydney University and the remaining 14 were recruited through word-of-mouth and social media advertisements. The participant group identified as culturally diverse (28 Australians, 6 Europeans, 14 Asians, 7 Middle Eastern, 1 Hispanic, 2 Aboriginals, 1 Native American and 1 American). Participation was respectively rewarded with course credit or $20. Thirty-four participants reported being able to play at least one musical instrument and 20 of those played for more than 5 years. Written informed consent was obtained from all participants prior to the start of the experiment, and the study was approved by the Western Sydney University Human Research Ethics Committee (H12632).

#### Materials

Stimuli were generated and displayed in MAX/MSP 7 (Cycling ‘74) in conjunction with Pianoteq 5 (v 5.8.1/20161125). The timbre of the tones was produced by Pianoteq 5 Pro (a high-quality physical synthesis model) with the instrument pack Grand D4 Daily Practice (Piano). Each trial presented consisted of a neutral musical context and a target triad. The context consisted of all thirteen individual notes of the chromatic BP scale in just intonation played twice and always in random order isochronously, immediate repetitions were not allowed, with an inter-onset interval (IOI) of 120 ms and at a MIDI key velocity of 64. The context was used to avoid effects of the previous trial on the next. The target triad followed 1000 ms after the context with a duration of 2000 ms and with a uniform MIDI key velocity value of 64. Triads were randomly selected from a subset of the 414 unique triads in BP just intonation. The chords were randomly transposed up in MIDI pitch until the 12th step of the BP scale. The lowest and the highest possible bass notes were 52 in MIDI pitch with a frequency of 164 Hz (no transposition) and 64 in MIDI pitch with a frequency of 329 Hz (highest transposition).

#### Procedure

The experiment was conducted in a soundproof room and participants were seated behind a laptop and listened through headphones. Headphones were either Sennheiser HD 280 Pro, Beyerdynamic DT 770 Pro or Sennheiser HD 650 depending on the availability at the time of the experiment. Volume was set at a comfortable level. First, the experimental task was explained. The experiment consisted of 4 blocks of 66 trials, so in total 264 trials. In two of the four blocks, participants were asked to rate the valence of the chord, i.e. how happy or sad the chord sounded on a continuous scale (presented as a horizontal slider) containing 700 discrete values invisible to the participant. The slider (“valence”) was labelled with the words “very unhappy” on the outer left side and “very happy” on the outer right side of the slider with the central point marked with a dash. In the two other blocks, participants rated the pleasantness (i.e. “consonance”) of the chord in a similar fashion. During those blocks, the labels of the slider were “very unpleasant” and “very pleasant”, with the central point marked with a dash. The order of the blocks was randomized. Small breaks in between the blocks were allowed and encouraged, but not required. Prior to the blocks, 5 practice trials were presented. At the end, participants filled out the Goldsmith’s Musical Sophistication Index (GMSI) questionnaire to record participants’ musical background. Three additional questions were added to check whether participants had absolute pitch, hearing problems and whether they were familiar with the sounds presented.

### Experiment 2

#### Participants

Sixty participants participated in experiment 2 (mean age = 25.9 years, SD = 9.15, 41 females). Participants included 36 undergraduate psychology students from Western Sydney University and the remaining 24 were again recruited through word-of-mouth and social media advertisements. The participant group identified as culturally diverse (35 Australians, 7 Europeans, 5 Asians, 8 Middle Eastern, 2 Hispanic, 1 American and 2 New Zealanders). Fourteen participants had participated in Experiment 1 as well, with a gap of two months between experiments. Participation was rewarded with either course credit or $20. Thirty-seven reported being able to play at least one musical instrument and 20 of those played for more than 5 years. Written informed consent was obtained from all participants prior to the start of the experiment, and the study was approved by the Western Sydney University Human Research Ethics Committee.

#### Materials

The generation of stimuli went through the exact same procedure as experiment 1, but the stimuli were this time presented in the equal temperament version of the BP scale.

#### Procedure

The procedure of Experiment 2 was analogous to the first experiment. Participants who had participated previously were told that the experiment was slightly different, but were not informed about the nature of the difference.

## Statistical methods

### Bayesian regression

In the Bayesian paradigm, probability is interpreted as a level of uncertainty, which is in contrast with the classical (frequentist) paradigm [58–59].

A multilevel Bayesian regression model was used to evaluate the effects of the independent variables on consonance and valence (i.e. pleasantness and happiness) ratings. Results were modelled and analysed in R [60] with the brms package using Stan [60–62]. The model used is the Bayesian version of a classical mixed effects model: it contains population-level effects (analogous to classical fixed effects) and group-level effects (analogous to classical random effects). In this case, the participants will be regarded as “group”, therefore the population-level effects are the estimated means of the effects across all participants tested; the group-level effects are the estimated standard deviations and correlations between the effects. An advantage of including group-level effects is that the model can account for differences between participants. For example, some participants may generally give lower ratings than others. Similarly, some participants may, for example, be relatively unfamiliar with Western music and so less sensitive to 12-TET Dissimilarity. At the same time, such a model can estimate the population-level effect of the “average” participant (given the pool of participants in the experiment). In comparison to models without group-level effects, models with group-level effects are less likely to provide evidence for non-existent effects (in non-Bayesian stats, this would be a Type I error) without sacrificing power [63].

The coefficients of a Bayesian regression have exactly the same meaning as in a standard regression: if the coefficient (effect) of predictor *k* is denoted *β_k_*, a unit increase in that predictor is associated with a *β_k_*-unit increase in the DV when all other predictors are held constant. A concise overview of Bayesian statistics in general is provided in Van de Schoot and Depaoli [59], whereas a more in-depth account can be found in Kruschke [64].

An important advantage of Bayesian regression is that, given the observed data and a prior distribution (see the next section for a discussion of priors), it calculates the whole posterior probability distribution of each effect’s size rather than only a point-estimate of the most probable effect size for each effect. A traditional power analysis, which assumes that a hypothesized parameter value is punctate, is in the Bayesian context therefore not applicable. This process allows for Bayesian credibility intervals to be calculated; unlike the confidence intervals in classical regression, credibility intervals have a straightforward and intuitive meaning: the 95% credibility interval of an effect size is the interval we can be 95% certain contains the effect’s true size.

Credibility intervals allow *evidence ratios* to be calculated; these are probability ratios (odds) in favour of directional hypotheses (such as a given effect being greater than zero): we term this directed hypothesis testing. For example, if the integral of the posterior distribution over the interval (0, ∞) is *p*, the evidence ratio in favour of the effect being greater than 0 is *p*/(1 –*p*); so, if the lower boundary of a (one-sided) 95% credibility interval is precisely zero, this implies there is a 5% probability the effect is less than zero and a 95% probability it is greater than zero, hence the evidence ratio is .95/.05 = 19.

To qualify the weight of evidence for or against any given hypothesis (e.g., that an effect is greater than 0), we follow the guidelines proposed by ([65] as cited by [66]): evidence ratios of 1–3 represent no evidence for the tested hypothesis; evidence ratios of 3–10 are “moderate” evidence for the hypothesis; evidence ratios of 10–30 are “strong” evidence; evidence ratios above 30 are “very strong” evidence. Evidence ratios close to zero represent evidence in favour of the null hypothesis.

### Priors

In Bayesian analyses, a prior distribution is chosen to reflect a parameter’s amount of (un)certainty before analysing the data [59]. A prior can, and should, reflect any prior knowledge, though as the amount of new data increases its influence in any case wanes. The priors used in all of our models are what are termed *weakly informative priors* [67–68] as there was little evidence about the consequences of the parameters included in the study. All of our non-binary predictors are standardized (they then have a standard deviation of 1; this suggests that we would be unlikely to see effect sizes with very high magnitudes).

For all population-level effects except the intercept, we used a prior with a Student’s *t*-distribution with 3 degrees of freedom, a mean of 0, and a scale of 2.5. Crucially, the zero mean indicates that our prior beliefs weakly favour the null hypothesis of zero effect size and–in comparison to using a flat uninformative prior–regularize the estimations, thereby reducing overfitting. Regularization is used to constrain a model’s flexibility in order to reduce uncertainty in the parameters by minimizing the *mean square error*.

### Models common to all experiments

The overall approach for analysing and modelling the results from both experiments is two-fold. First, a *descriptive model* of mean ratings per chord type and average pitch height (and average pitch height- squared) is fitted, to provide essential data visualization and a summary of the data. Second, a *predictive model* (a mixed-effects model) of the evaluative ratings with the intrinsic and extrinsic predictors outlined above is fitted to estimate the strengths of the proposed underlying factors. This procedure is done for Experiment 1 for both *Consonance* and *Valence*, subsequently for Experiment 2 and lastly, for the two experiments combined. In the interests of brevity, only the ratings and the predictive models for the two experiments combined will be reported in the main text. The separate analyses can be found in the supplementary materials.

Average chord ratings for each response variable were computed with rating as dependent variable and chord number (as factor), with average pitch height and average pitch height squared as independent variables.

Chord numbers were turned into factors and Chord 0 6 10 (numbers indicate BP semitone steps) was set as intercept. Chord 0 6 10 is the theoretical BP major chord and therefore might have the highest consonance and valence ratings, but the experimental results from Mathews et al. [26] suggest otherwise. Chord ratings should therefore be interpreted with respect to this chord.

The pleasantness and valence ratings were separately modelled by the previously discussed intrinsic and extrinsic predictors: roughness (Roughness), harmonicity (Harmonicity), spectral entropy (SpectralEntropy), 12-TET Dissimilarity (12-TET Dissimilarity), average pitch height (AveragePitch) and average pitch height squared (AveragePitch^2), each of these interacting with musical sophistication (GMSI). All of the predictors were included as random effects. Stolzenburg’s periodicity measure [69] was tested as an alternative to harmonicity and was found–using leave-one-out cross-validation–to perform slightly worse at predicting out-of-sample data. All predictors and the ratings were standardized and scaled to their means except for Goldsmiths Musical Sophistication Index (GMSI), which was centred to the previously determined population mean from Müllensiefen et al. [57] (see Section *Musical Sophistication*). The 700 discrete values obtained from the slider ratings were modelled as being normally distributed, an assumption borne out by the posterior predictive checks shown in the supplementary materials. All the models converged as evidenced by Rhat. values below 1.

## Results

### Just intonation and equal temperament

Two sets of participants have been exposed to two different tunings of the BP system, in contrast with the original study by Mathews et al. [26] who solely tested the equal tempered version of the scale. In the just intonation version of the scale, we tested 414 chords due to the slightly unequal sizing of the 13 BP semitone steps. Before analysing whether participants perceived one tuning differently from the other, two models were fitted in order to find out whether grouping the 414 chords based on each different chord tuning into the 66 categories based purely on the chord’s step-size intervals was plausible. To clarify, by comparing these two models it is examined whether the 414 stimuli can be categorized into 66 units rather than 414. Both models included covariates of Average Pitch Height and Average Pitch Height-squared and all chord categories were referenced to Chord 0 6 10. This procedure was conducted for both the Consonance and Valence data. Approximate leave-one-out cross-validation was performed with LOO from the brms package [60–62] and it was demonstrated the simpler (66 grouping) model had a smaller LOO number than the more complex (414 grouping) model (shown in Table 3) suggesting a better fit. This suggests that grouping the 414 chords in 66 categories is credible, and therefore only the best fitting model (with 66 chord groups) is presented for Experiment 1.

**Table 3:** Testing whether the just intonation chords can best be considered as 414 items or 66 groups using the LOO Information Criterion.

	*LOOIC*
Consonance414	7716.61
Consonance66	7632.18
Consonance414 -Consonance66	84.43
Valence414	6201.39
Valence66	6020.22
Valence414-Valence66	181.17

*Notes*. Consonance414 and Valence 414 represent the consonance and valence models with 414 chords, respectively. Consonance 66 and Valence66 represent the models with 66 chords, respectively. The table shows LOOIC (LOO Information Criterion).

After this procedure, it was analysed whether ratings in equal temperament differed from those in just intonation. Two models were fitted to compare consonance ratings in both tunings and valence ratings in both tunings with the chord ratings and tuning as categorical variables. There was no effect of tuning on the ratings, as the 95% Bayesian credibility intervals for tuning included zero. Therefore, we collapsed the two experiments and present the combined models of both.

An exploratory regression was conducted in order to estimate whether short-term statistical learning had taken place throughout the experiment, as such results have been previously found by Loui [32,33]. Besides, it helps to determine whether participation in related experiments affected ratings. TrialNo (accumulative trial numbers), TrialNo^2 (squared to allow for nonlinearity in the effects), ChordNo (the number of times participants have heard a particular chord) and ChordNo^2 were included as independent variables to test their effects on consonance and valence ratings. The resulting models are reported in the supplementary materials; in summary, predictors whose credibility intervals do not cross zero are reported here. TrialNo had a small effect on consonance ratings (= -.04). Small effects for valence ratings were found for ChordNo (= 0.11) and ChordNo^2 (= -.02). These results indicate that consonance ratings slightly decreased with an increase in trial number, which might suggest the presence of a boredom effect. Valence ratings went up slightly with an increase in chord number, which hints towards a subtle mere exposure effect. These weak effects do not suggest strong learning during the experiment, therefore participants who have participated in both will be included in the analyses.

### Descriptive models of consonance and valence ratings

**Fig 1:**
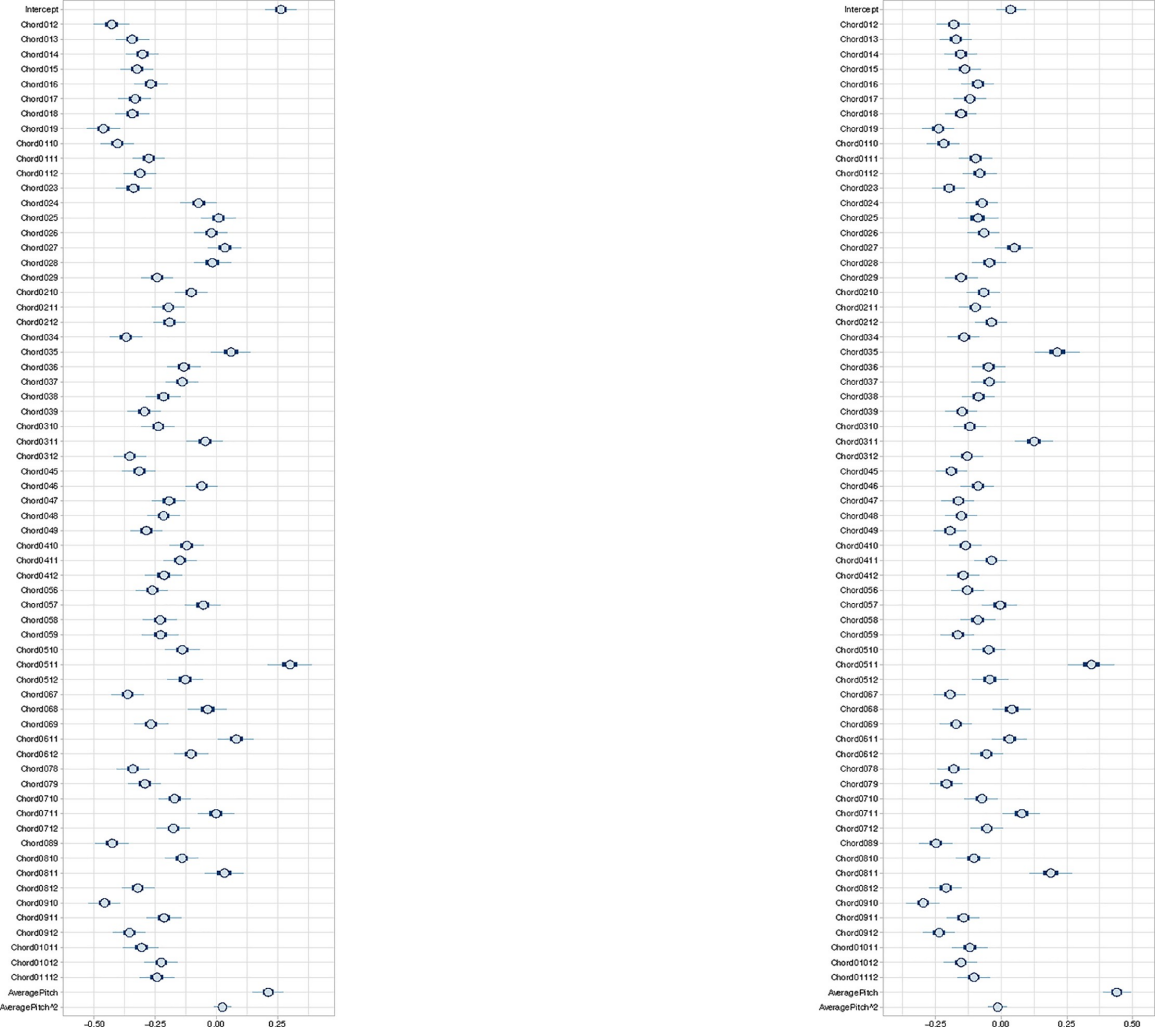
Mean consonance (left) and valence (right) ratings with 95% credibility intervals after controlling for a quadratic function of average pitch height. The thick darker blue line shows the 50% interval and the thinner light blue line shows the 95% interval.

Fig 1 shows the mean ratings for the 66 chords for consonance (left) and valence (right) for both experiments combined, and the effects of the predictors AveragePitch and AveragePitch^2. The model accounted for autoregressive effects of each participant’s previous response on their next response. The credibility intervals (illustrated by the thin blue lines) show the range of values within which there is a 95% probability that the observed ratings’ mean values fall. Average Pitch seems to have an effect on the overall ratings of the chords, suggesting that the higher the transposition of a chord, the more pleasant/happy it is rated. The size of the difference between the most consonant and least consonant is large (mean = 0.76). For valence, the size of the effect between the happiest and the least happy chord is also large (mean = 0.64). Importantly, the majority of responses are below zero, which indicates that the ratings are generally lower than for the reference item.

Marginal effect plots of Average Pitch Height and histograms depicting the mean ratings across the 66 chords for Experiment 1 and 2 separately and combined can be found in the supplementary materials.

**Fig 2:**
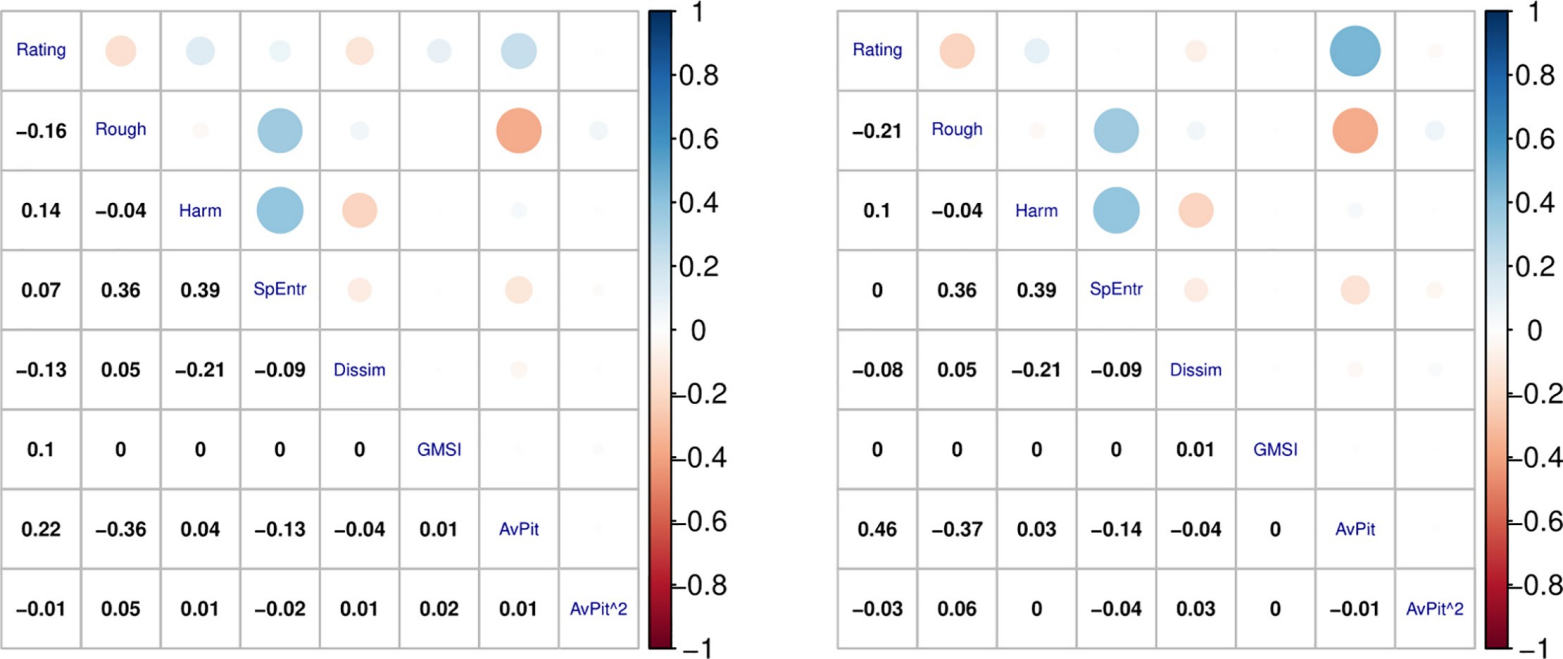
Correlation matrix showing correlations between consonance ratings (left), valence ratings (right) and Roughness, Harmonicity, Spectral Entropy, 12-TET Dissimilarity, GMSI, Average Pitch, and Average Pitch^2.

Correlational analyses were conducted to examine the relationship between the different predictors for consonance and valence ratings (Fig 2). For consonance, moderate positive linear relationships were found between Roughness and Spectral Entropy (r = .36), Harmonicity and Spectral Entropy (r = .39), and a moderate negative relationship was found for Roughness and Average Pitch (r = -.36). For valence, moderate positive linear relationships were found between Roughness and Spectral Entropy (r = .36), Harmonicity and Spectral Entropy (r = .39), Rating and Average Pitch (r = .41) and a moderate negative relationship was found for Roughness and Average Pitch (r = -.37).

### Predictive model of consonance

**Fig 3:**
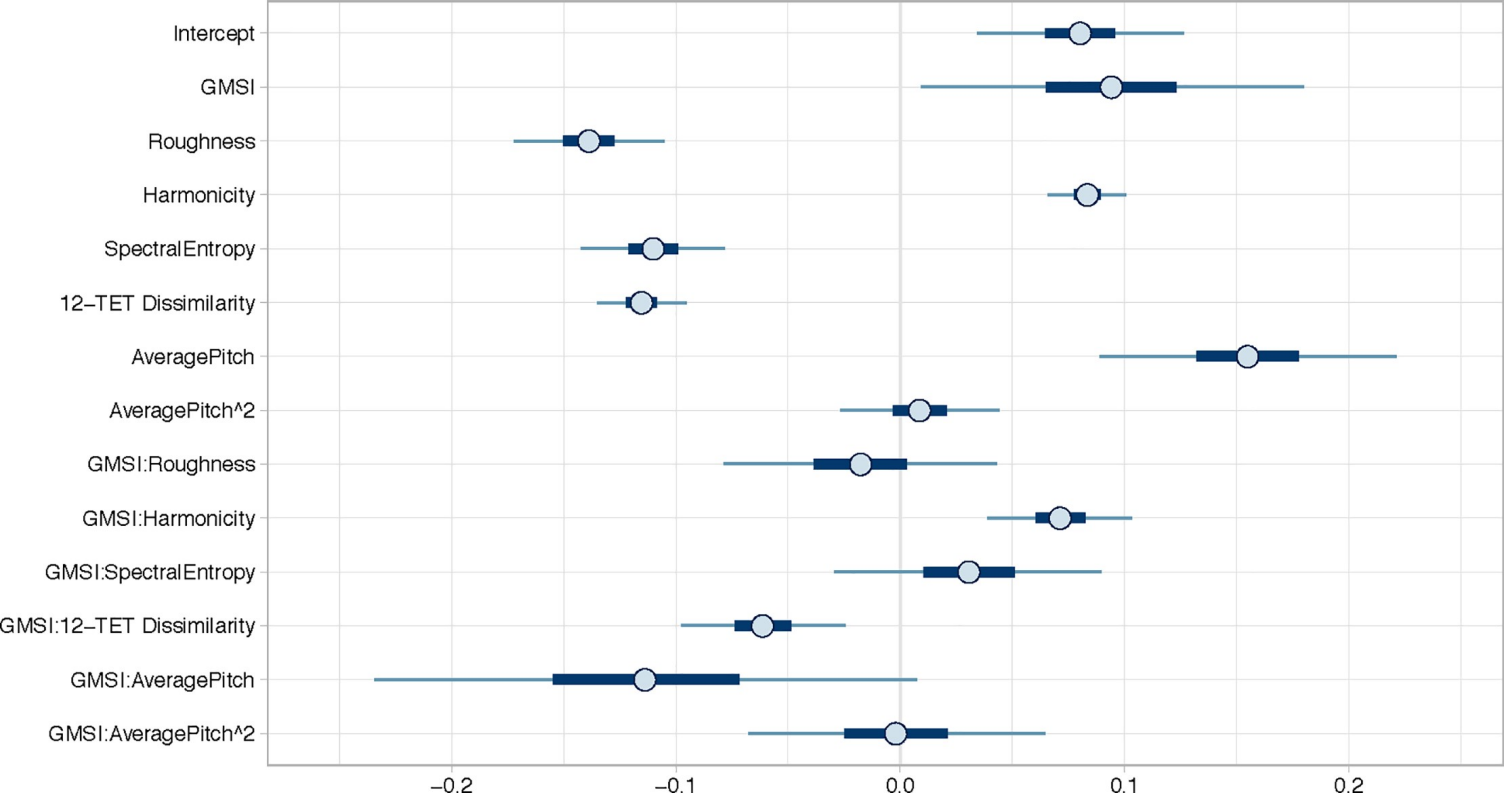
Consonance model with all modelled parameters and 95% Bayesian credibility intervals for Experiment 1 and 2. The thick darker blue line shows the 50% interval and the thinner light blue line shows the 95% interval.

Fig 3 shows the 95% Bayesian credibility intervals of the predictor variables for a model of Consonance for both experiments combined. The predictors were also modelled for the experiments separately, but the results were very similar showing that the predictors generalize well between the subtle tuning differences and that they are consistent across experiments. For that reason, we focus here on both experiments combined (separate analyses of Experiment 1 and 2 can be found in the supplementary materials). As hypothesized, Roughness, Harmonicity, Spectral Entropy, 12-TET Dissimilarity and Average Pitch Height all predict consonance in the expected direction. The evidence ratios for the conditional main effects of the predictors are all “very strong”, as presented in Table 4. Interesting to note is that there is also evidence to suggest that participants with more musical training gave higher pleasantness ratings. Strong evidence was obtained for interactions between Goldsmith’s Musical Sophistication Index (GMSI) and Harmonicity and GMSI and 12-TET Dissimilarity. The coefficients are small for Roughness (= -.14), Spectral Entropy (= -.11), 12-TET Dissimilarity (= -.12), Average Pitch (= .15) and GMSI:AveragePitch (= .11). The results of the Bayesian regression model indicated that the predictors explained 41.6% of the variance in the data.

**Table 4:** Directed hypothesis testing for consonance.

Hypothesis	Estimate	Est. Error	CI Lower	CI Upper	Evid. Ratio	Star
Roughness < 0	-0.14	0.02	-Inf.	-0.11	Inf.	*
Harmonicity > 0	0.08	0.01	0.07	Inf.	Inf.	*
SpectralEntropy < 0	-0.11	0.02	-Inf.	-0.08	Inf.	*
12-TET Dissimilarity < 0	-0.12	0.01	-Inf.	-0.10	Inf.	*
AveragePitch > 0	0.15	0.03	0.10	Inf.	Inf.	*
AveragePitch^2 > 0	0.03	0.01	0.01	Inf.	Inf.	*
GMSI:Roughness < 0	-0.02	0.03	-Inf.	0.03	2.55	
GMSI:Harmonicity > 0	0.07	0.02	0.04	Inf.	Inf.	*
GMSI:SpectralEntropy < 0	0.03	0.03	-0.02	Inf.	5.51	
GMSI:12-TET Dissimilarity > 0	-0.06	0.02	-Inf.	-0.03	1499.00	*
GMSI:AveragePitch < 0	-0.11	0.06	-Inf.	0.01	29.46	*
GMSI:AveragePitch^2 > 0	0.02	0.02	0.00	Inf.	Inf.	*

* = The expected value under the hypothesis lies outside the 95%-CI (credibility intervals). Estimate = mean.

Estimate error = standard deviation of the posterior distribution. CI lower and CI upper are two-sided 95% credibility intervals. Evidence ratio = the posterior probability under the hypothesis against its alternative.

**Fig 4:**
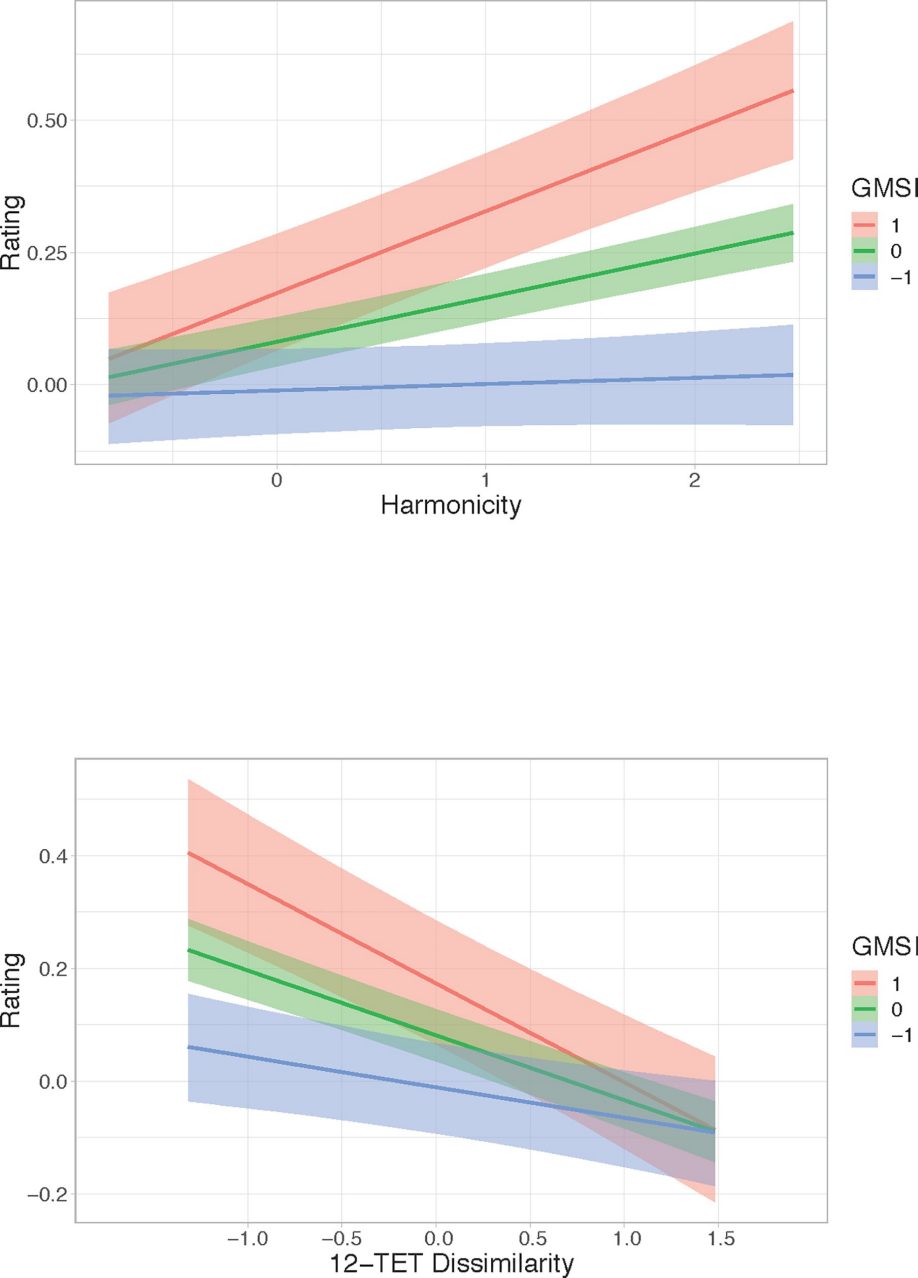
Marginal effects plots for GMSI and Harmonicity and GMSI and 12-TET Dissimilarity. Levels of GMSI represent the mean (0), 1 SD above the mean (1) and 1 SD below the mean (-1).

The marginal effects plots in Fig 4 show the impact of the moderating effect of GMSI on Harmonicity and 12-TET Dissimilarity. GMSI does not interact with the effects of the other predictors.

The Harmonicity plot shows that, as hypothesized, chords with higher levels of harmonicity are perceived as more pleasant. Furthermore, this effect is stronger for musically sophisticated participants than it is for musically unsophisticated participants. The 12-TET Dissimilarity plot illustrates that the more distant a chord is from 12-TET, the less pleasant it is (this is as hypothesized); again, this effect is stronger for musically sophisticated participants than for musically unsophisticated participants.

### Predictive model of valence

**Fig 5:**
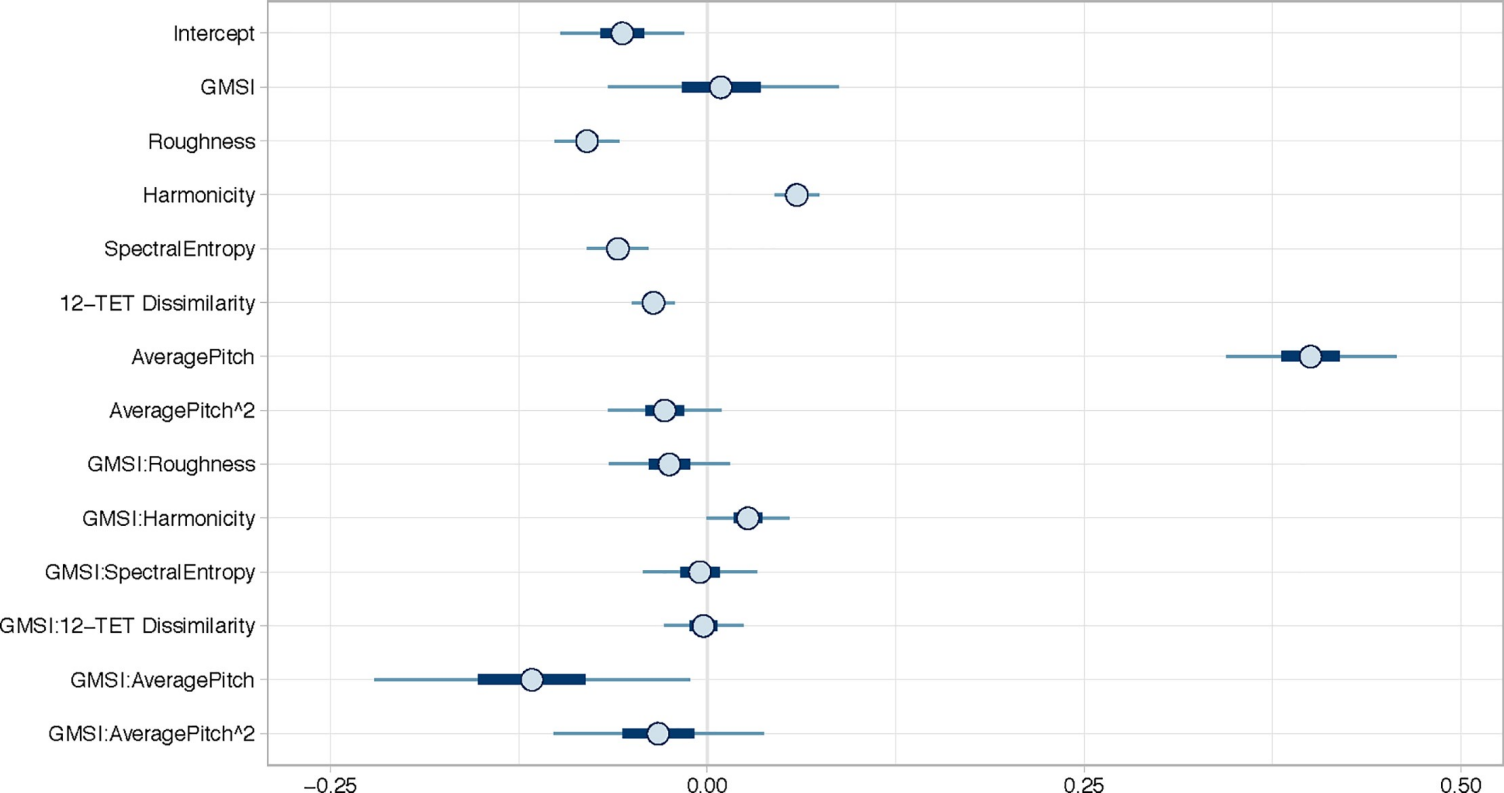
Valence model with all modelled parameters and 95% Bayesian credibility intervals for Experiment 1 and 2. The thick darker blue line shows the 50% interval and the thinner light blue line shows the 95% interval.

Fig 5 shows the 95% Bayesian credibility intervals of the independent variables for Valence for both experiments combined. As hypothesized, Roughness, Harmonicity, Spectral Entropy, 12-TET Dissimilarity and Average Pitch Height all predict consonance in the expected direction. The evidence ratios for the conditional main effects of the predictors are all “very strong”, as presented in Table 5. Strong evidence was obtained for interactions between GMSI and Harmonicity and between GMSI and Average Pitch. The coefficient for Average Pitch is medium (= .4) and small for GMSI:Average Pitch (= -.12). Smaller coefficients are found for the other predictors, namely Roughness (= -.08), Harmonicity (= .06), Spectral Entropy (= -.06) and 12-TET Dissimilarity (= -.04). The effect of Average Pitch Height is quite striking as it is very strong in comparison to the other parameters and to its effect on the consonance ratings. The results of the Bayesian regression model indicated that the predictors explained 47.4% of the data.

**Table 5:** Directed hypothesis testing for valence.

Hypothesis	Estimate	Est. Error	CI Lower	CI Upper	Evid. Ratio	Star
Roughness < 0	-0.08	0.01	-Inf.	-0.06	Inf.	*
Harmonicity > 0	0.06	0.01	0.05	Inf.	Inf.	*
SpectralEntropy < 0	-0.06	0.01	-Inf.	-0.04	Inf.	*
12-TET Dissimilarity < 0	-0.04	0.01	-Inf.	-0.02	Inf.	*
AveragePitch > 0	0.40	0.03	0.35	Inf.	Inf.	*
AveragePitch^2 > 0	0.16	0.02	0.12	Inf.	Inf.	*
GMSI:Roughness < 0	-0.03	0.02	-Inf.	0.01	8.11	
GMSI:Harmonicity > 0	0.03	0.01	0.00	Inf.	38.13	*
GMSI:SpectralEntropy < 0	0.00	0.02	-0.04	Inf.	0.68	
GMSI:12-TET Dissimilarity > 0	0.00	0.01	-Inf.	0.02	1.34	
GMSI:AveragePitch < 0	-0.12	0.05	-Inf.	-0.03	64.69	*
GMSI:AveragePitch^2 > 0	0.02	0.01	0.00	Inf.	Inf.	*

* = The expected value under the hypothesis lies outside the 95%-CI (credibility intervals). Estimate = mean.

Estimate error = standard deviation of the posterior distribution. CI lower and CI upper are two-sided 95% credibility intervals. Evidence ratio = the posterior probability under the hypothesis against its alternative.

**Fig 6:**
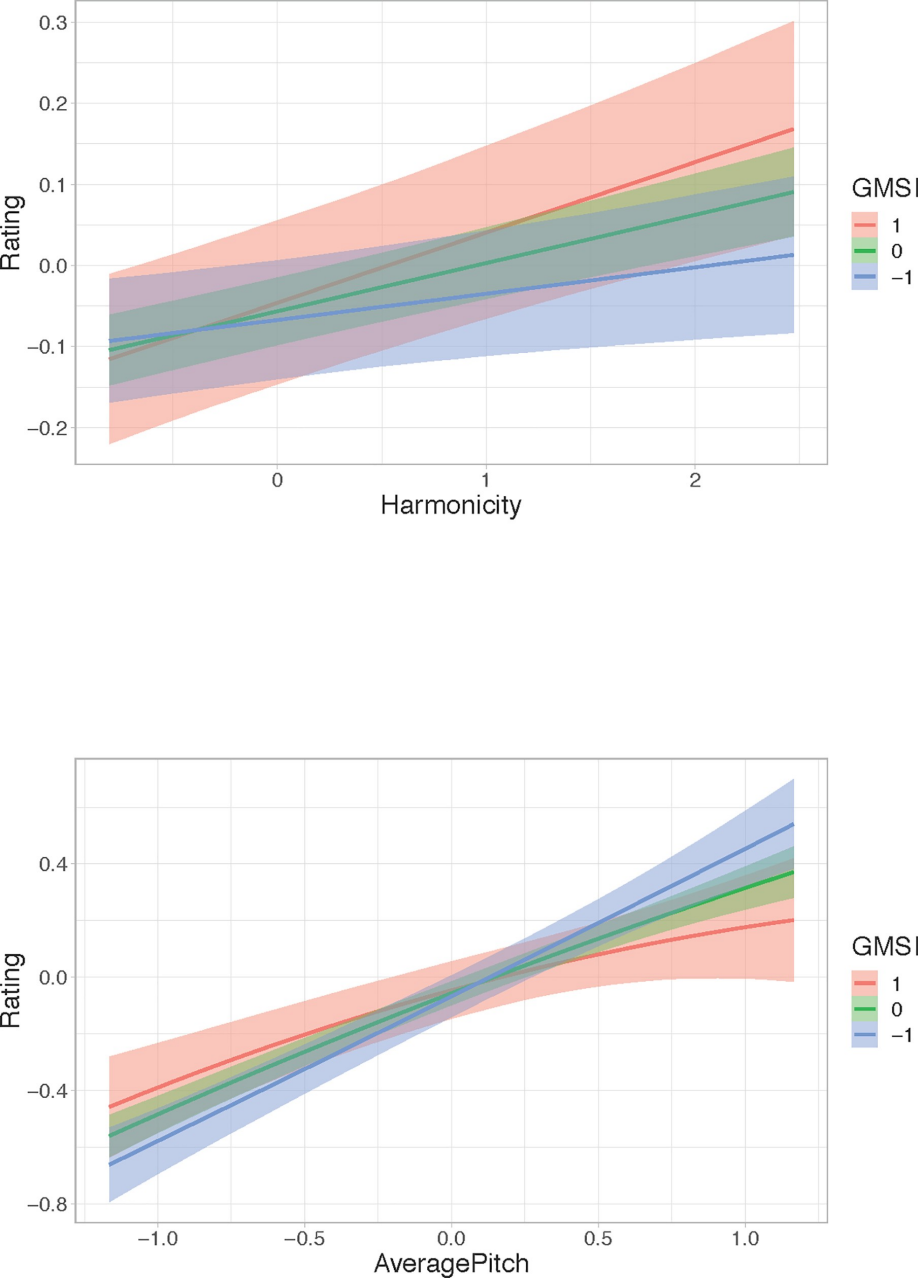
Marginal effects plots for GMSI and Harmonicity and GMSI and Average Pitch. Levels of GMSI represent the mean (0), 1 SD above the mean (1) and 1 SD below the mean (-1).

Fig 6 shows the marginal plots for the interaction between GMSI and Harmonicity and between GMSI and Average Pitch. This interaction shows a similar pattern as the previously discussed interaction between GMSI and Harmonicity in the Consonance model. Chords with higher harmonicity levels were rated happier by participants with a high GMSI.

Kendall’s rank correlation was conducted to see whether the consonance and valence ratings are associated. Based on the results of this study, consonance ratings are independent from valence ratings *r*_*τ*_ = .14, *p <* .05. Out of the 66 chords, Bonferroni corrected dependent Wilcoxon signed-rank tests demonstrated that a total of 7 chords were rated significantly differently in the consonance versus the valence condition (*p <* .05).

## Discussion

The current study examined the influence of intrinsic features on affective responses to unfamiliar musical stimuli. In two experiments, participants rated the consonance and valence of chords from the Bohlen-Pierce system, and a set of intrinsic features (roughness, harmonicity, spectral entropy and average pitch height) and one extrinsic feature (familiarity) were used to predict their responses. Roughness was chosen as a well-established, but somewhat disputed, predictor of music affect. Harmonicity is a familiar concept, but has a variety of mathematical formulations; it was calculated here in a novel way using expectation tensors. Spectral entropy is usually a predictor of a proposed timbral dimension, and has never been used as a predictor of consonance. 12-TET Dissimilarity is a straightforward but novel predictor, and average pitch height and average pitch height squared complete the set of predictors used. All these predictors were combined with musical sophistication as assessed by the Goldsmiths Musical Sophistication Index. By using such a large set of both novel and established predictors, a systematic approach was taken, estimating the independent effects of each variable and how it is moderated by musical sophistication.

The results from both experiments were consistent and suggest the following notable findings: (1) consistently across participants, some chords from the BP system have distinctly different ratings of consonance and valence; (2) the subtle changes between the two tuning systems did not have an impact on participants’ ratings; (3) the two predictive models for consonance and valence are quite similar, apart from the effect of Average Pitch height, which differs in degree rather than direction; (4) there is very strong evidence that all of the intrinsic predictors in the models are independently correlated with the ratings as hypothesized.

### Chord ratings

Similar to the original study of Mathews et al. [26], a large range of consonance was observed. Since we did not include the tritave in the chord selection as was done in Mathews et al. [26], a direct comparison between the most consonant and dissonant chords cannot be made, but several chords do appear to have strong ratings in both studies, for example Chords 0 6 10 (the BP major chord), 0 5 11, 0 3 5 and 0 8 11. Note that in their study, results were split into two participant groups, namely musicians and non-musicians. Chord 0 5 11 and 0 3 5 were among the highest rated chords for musicians, whereas Chord 0 6 10 and 0 8 11 scored high in the non-musician group. An important implication that can be drawn from the chord ratings overall, from both this study and Mathews et al.’s study [26], is that *previously unheard* BP chords are rated *consistently* across participants. Furthermore, the highest and the lowest rated chords are consistent across participants. Specifically, Chord 0 5 11 received very high ratings for both consonance and valence in both experiments. Can this be explained by any of the intrinsic factors or is this purely caused by its intervals being of close proximity to Western intervals? Several high and low rated chords and their respective Roughness, Harmonicity, 12-TET Dissimilarity and Spectral Entropy values, as well as descriptive statistics for those predictors, are presented in Tables 6–8. For the highest rated chords, Chord 0 5 11 reaches a harmonicity value more than 1 standard deviation higher than the mean and Chord 0 8 11 is close to that as well. Apart from Chord 0 3 11 and 0 6 11, all other chords have harmonicity values in the third quartile, and exemplify the role of harmonicity for the perception of positive affect. Spectral Entropy, 12-TET Dissimilarity and Roughness values do not show a clear pattern, suggesting that the high ratings obtained for these chords are not due to those factors.

**Table 6:** Descriptive statistics for roughness, harmonicity, spectral entropy, and 12-TET dissimilarity.

	Roughness	Harmonicity	Spectral Entropy	12-TET Dissimilarity
Minimum	-0.69	-0.60	-1.31	-1.10
1^St^ Quartile	-0.41	-0.33	-0.33	-0.35
Median	-0.12	-0.12	0.13	-0.09
Mean	0.00	0.00	-0.00	-0.00
3^rd^ Quartile	0.28	0.09	0.25	0.34
Maximum	1.58	2.16	0.77	1.20

*Notes*. Values are standardized.

**Table 7:** Roughness, harmonicity, spectral entropy and 12-TET dissimilarity values for several high rated chords for consonance and valence.

	Roughness	Harmonicity	Spectral Entropy	12-TET Dissimilarity
Chord 0 3 5	0.54	2.16	0.35	0.08
Chord 0 3 11	-0.34	-0.25	0.18	-0.33
Chord 0 5 11	-0.54	1.18	0.12	-0.27
Chord 0 6 11	-0.54	0.00	-0.17	-0.90
Chord 0 8 11	-0.05	0.92	-0.35	-0.33
Chord 0 6 10 (Intercept)	-0.44	2.16	-0.29	0.08

*Notes*. Values are standardized.

**Table 8:** Roughness, harmonicity, spectral entropy and 12-TET dissimilarity values for several low rated chords for consonance and valence.

	Roughness	Harmonicity	Spectral Entropy	12-TET Dissimilarity
Chord 0 1 2	1.58	0.19	-0.27	-0.33
Chord 0 1 9	0.23	-0.50	-0.08	0.19
Chord 0 1 10	0.04	-0.30	-0.78	0.60
Chord 0 8 9	0.04	-0.36	-0.30	0.25
Chord 0 9 10	-0.17	0.03	0.50	0.19
Chord 0 6 10 (Intercept)	-0.44	2.16	-0.29	0.08

*Notes*. Values are standardized.

Consideration of the lowest rated chords in Table 8, reveals a maximum Roughness value is obtained for Chord 0 1 2. However, this chord was only among the lowest rated chords for Consonance, but not for Valence. Spectral entropy is quite high for Chord 0 9 10 in comparison with the highest rated chords. The pattern among the predictors is less obvious for the lowest-rated chords. This could mean that perception of consonance and valence is a more complex process involving a combination of the factors tested in this study, nonlinearities in their effects, or perhaps other predictors that have not been modelled.

### Intrinsic and extrinsic predictors

Apart from demonstrating that unfamiliar chords can evoke different affective responses, this study has provided very strong evidence that all the predictors have an effect in the expected direction. Furthermore, the effects are consistent across the two experiments despite slightly different tunings, and broadly consistent across the two affective responses. They are also consistent in direction across musical expertise. This study provides strong evidence that intrinsic features influence affective responses–consonance and valence–to music. To reiterate, intrinsic features were defined as features that do not rely on long-term statistical regularities and that have the potential to function across different musical cultures.

The role of roughness and harmonicity in consonance perception has been thoroughly tested and criticized over the years in numerous theoretical and experimental studies (e.g. [35,3–5,17, 70–71], but there are many more). Roughness was found to be negatively correlated with pleasantness and happiness ratings, and this effect was not moderated by musical sophistication. This suggests, in contrast to McLachlan et al. [38] that perception of roughness in unfamiliar chords is not affected by a musical training effect.

Similar to findings of McDermott et al. [43], an interaction was found in both experiments between harmonicity and musical sophistication in predicting consonance and in predicting valence ratings in the second experiment. Causal explanations for this moderation include the possibility of musical experience amplifying preferences for harmonicity (as suggested by McDermott et al. [43]) or that people who are sensitive to harmonicity are drawn to music and hence have higher musical sophistication. The latter might, however, suggest a similar moderation effect occurring for roughness, for which there was only limited evidence in our data.

Spectral entropy is a novel predictor for the consonance and valence of chords. It was found to be a useful predictor, in the expected direction in all models tested, both for consonance and valence. As mentioned earlier, spectral entropy is a way to quantify the unpredictability of the spectrum of the chord. An implication of a highly unpredictable chord spectrum is that it will evoke uncertainty about which pitches are to be perceived or how these fit to a harmonic series [70]. Complex and unpredictable spectra are more difficult to perceive and process. A chord with high spectral entropy could therefore negatively affect processing fluency which would subsequently lead to a more negative aesthetic experience (such as the perception of dissonance).

Another notable finding of this study is the strong effect of average pitch height in both models, supporting the theory that high pitch is associated with positive affect, with positive applying to both pleasantness and happiness [28,51–52,31]. These correlations may partly be explained by the relationship between music and vocal affect expression [35,72–73]. The strong effect of pitch height on valence ratings is not surprising, as pitch height is often associated with happiness in music [28,51–52,31], but interestingly, consonance ratings were also partially predicted by pitch height. However, the strength of pitch height on valence responses was considerably larger than on consonance responses.

Possibly, pitch height could be interpreted being a musical ‘universal’ as those with lower levels of musical sophistication nonetheless instantly recognize it as happy. An interaction between musical sophistication and average pitch was only found in the valence model. Even though it is a fairly small effect, it was shown that less musically sophisticated participants were more affected by a higher average pitch.

A limitation of this study is the over-representation of first-year psychology students in the participant pool. Even though a separate recruitment procedure was conducted, the overall majority of the participants were recruited as part of their psychology course requirements, resulting in limitations in, for example, age diversity. However, the multicultural community that can be found at Western Sydney University ensured a culturally and linguistically diverse participant pool.

Also, this study examined a specific set of theoretically motivated intrinsic predictors while including only a single extrinsic predictor because of the unfamiliar nature of the stimuli. Although the study showed that this specific set of predictors have an impact on affective responses to music, the Bayesian R-squared of .41 for consonance and .47 for valence show that the majority of variance remains unexplained; for this reason, it would be interesting and useful to develop additional new predictors.

## Conclusion

This study aimed to present a systematic approach to examine perception of consonance and valence of an unfamiliar musical system. A number of novel and established intrinsic and extrinsic predictors were mathematically modelled. The results and models have given novel insights into how consonance and valence are perceived in the simple musical elements of chords. Crucially, we obtain strong evidence that a number of features intrinsic to an unfamiliar musical system impact on affective responses.

Similar to Milne et al. [42], effects of interval familiarity were minimized through the experimental design and the use of an unfamiliar microtonal system. Therefore, the effects of the intrinsic predictors found on consonance and valence ratings can be credibly attributed to the musical stimuli themselves, rather than to familiarity or learned associations. Altogether, the results indicate that, in an unfamiliar musical system, it is possible to show the important influence of intrinsic predictors on the perception of consonance and valence. This is in contrast with much current research in music perception, which argues that extrinsic or learned processes are of more importance than intrinsic predictors (e.g. [15–16]). Our results also suggest promising directions for future research in the field of music perception and affect: by moving away from commonly used Western musical stimuli, we can show that–in addition to long-term statistical learning of a musical system and its cultural context–fundamental perceptual mechanisms are at the heart of musical communication. The extent to which experience with a musical system might supersede these influences remains to be determined. Future experiments will consider further extrinsic aspects such as the effect of manipulating exposure to the system and a reductive causal intervention experiment including continuous affective responses to longer fragments of music in a Western tuning and a Bohlen-Pierce tuning.

## Supporting information

S1 Fig Consonance (left column) and Valence (right column) for Experiment 1 (top), Experiment 2 (middle) and Experiment 1&2 combined (bottom).
(TIFF)

S2 Fig Consonance (left column) and Valence (right column) for Experiment 1 (top), Experiment 2 (middle) and Experiment 1&2 combined (bottom). The histograms show whether ratings are skewed or normal in their distribution.
(TIFF)

S3 Fig Consonance (left column) and Valence (right column) for Experiment 1 (top), Experiment 2 (middle) and Experiment 1&2 combined (bottom). The thin black line is the distribution of the observed outcomes and the blue lines represent the 1000 draws from the posterior predictive distribution.
(TIFF)

S4 Fig Mean consonance (left) and valence (right) ratings with 95% credibility intervals after controlling for a quadratic function of average pitch height. The thick darker blue line shows the 50% interval and the thinner light blue line shows the 95% interval.
(TIFF)

S5 Fig Consonance model with all modelled parameters and 95% Bayesian credibility intervals for Experiment 1. The thick darker blue line shows the 50% interval and the thinner light blue line shows the 95% interval.
(TIFF)

S6 Fig Marginal effects plots for GMSI and Harmonicity and GMSI and 12-TET Dissimilarity. Levels of GMSI represent the mean (0), 1 SD above the mean (1) and 1 SD below the mean (-1).
(TIFF)

S7 Fig Valence model with all modelled parameters and 95% Bayesian credibility intervals for Experiment 1. The thick darker blue line shows the 50% interval and the thinner light blue line shows the 95% interval.
(TIFF)

S8 Fig Mean consonance (left) and valence (right) ratings with 95% credibility intervals after controlling for a quadratic function of average pitch height. The thick darker blue line shows the 50% interval and the thinner light blue line shows the 95% interval.
(TIFF)

S9 Fig Consonance model with all modelled parameters and 95% Bayesian credibility intervals for Experiment 2. The thick darker blue line shows the 50% interval and the thinner light blue line shows the 95% interval.
(TIFF)

S10 Fig Marginal effects plots for GMSI and Harmonicity and GMSI and 12-TET Dissimilarity. Levels of GMSI represent the mean (0), 1 SD above the mean (1) and 1 SD below the mean (-1).
(TIFF)

S11 Fig Valence model with all modelled parameters and 95% Bayesian credibility intervals for Experiment 2. The thick darker blue line shows the 50% interval and the thinner light blue line shows the 95% interval.
(TIFF)

S12 Fig Marginal effects plot for GMSI and Harmonicity. Levels of GMSI represent the mean (0), 1 SD above the mean (1) and 1 SD below the mean (-1).
(TIFF)

S1 File Supplementary materials. (DOCX)
