# Supplementary figures and images for: Perception of affect in unfamiliar musical chords
Source: PLoS One. 2019 Jun 21;14(6):e0218570. doi: 10.1371/journal.pone.0218570 (PMC6588276; doi:10.1371/journal.pone.0218570)

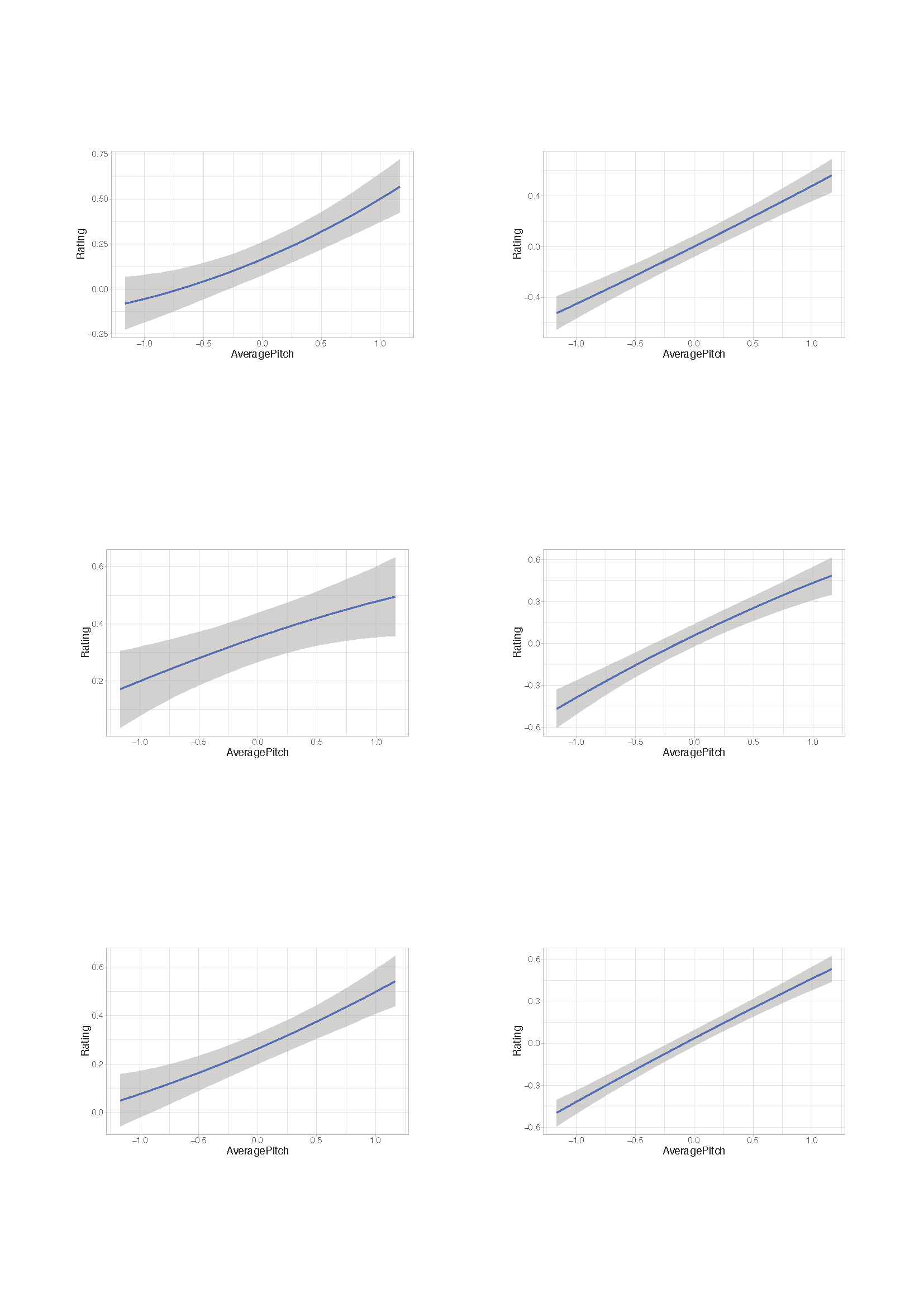

Supplement: S1 Fig — Consonance (left column) and Valence (right column) for Experiment 1 (top), Experiment 2 (middle) and Experiment 1&2 combined (bottom). (TIFF) [file pone.0218570.s001.tiff]

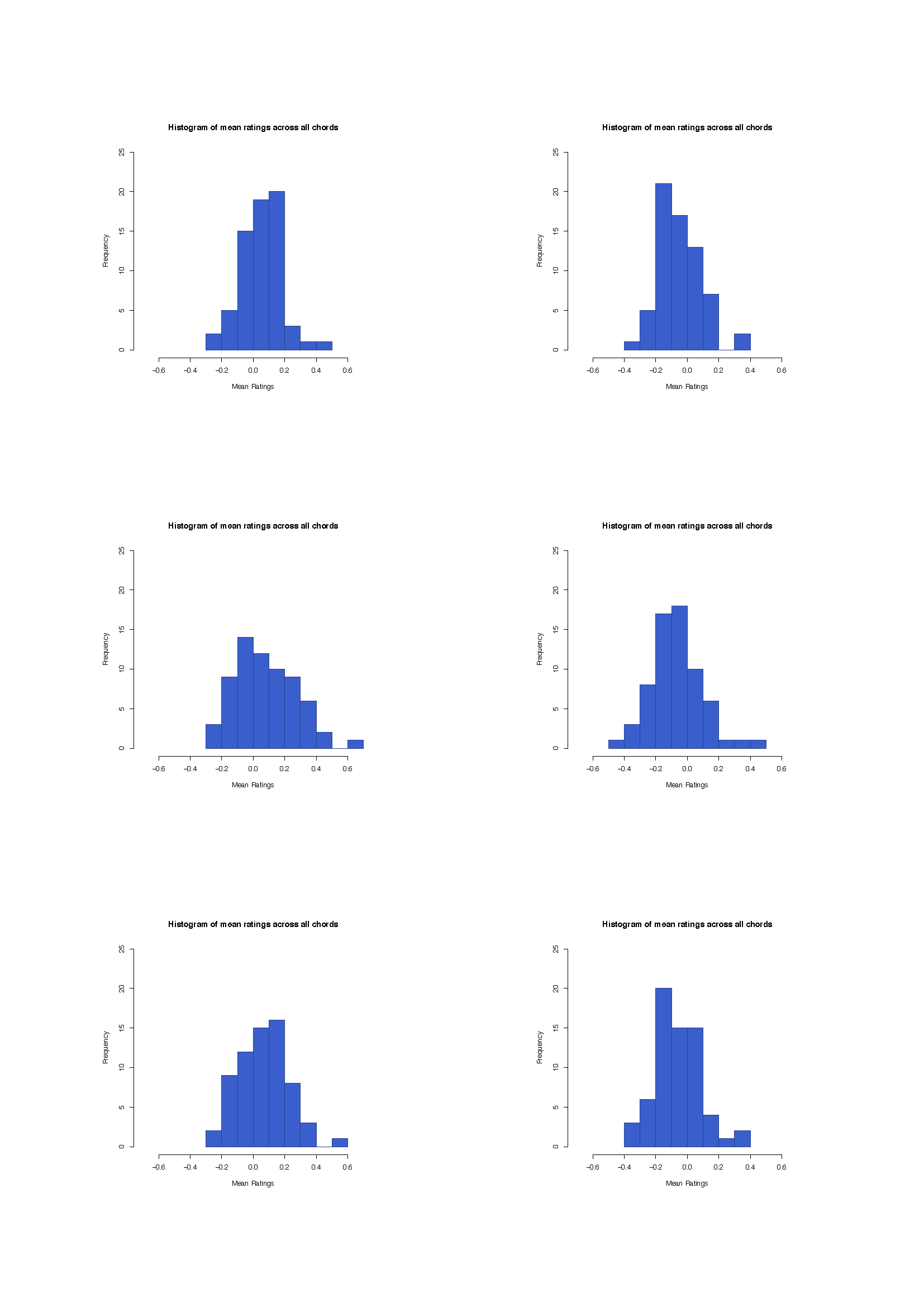

Supplement: S2 Fig — Consonance (left column) and Valence (right column) for Experiment 1 (top), Experiment 2 (middle) and Experiment 1&2 combined (bottom). The histograms show whether ratings are skewed or normal in their distribution. (TIFF) [file pone.0218570.s002.tiff]

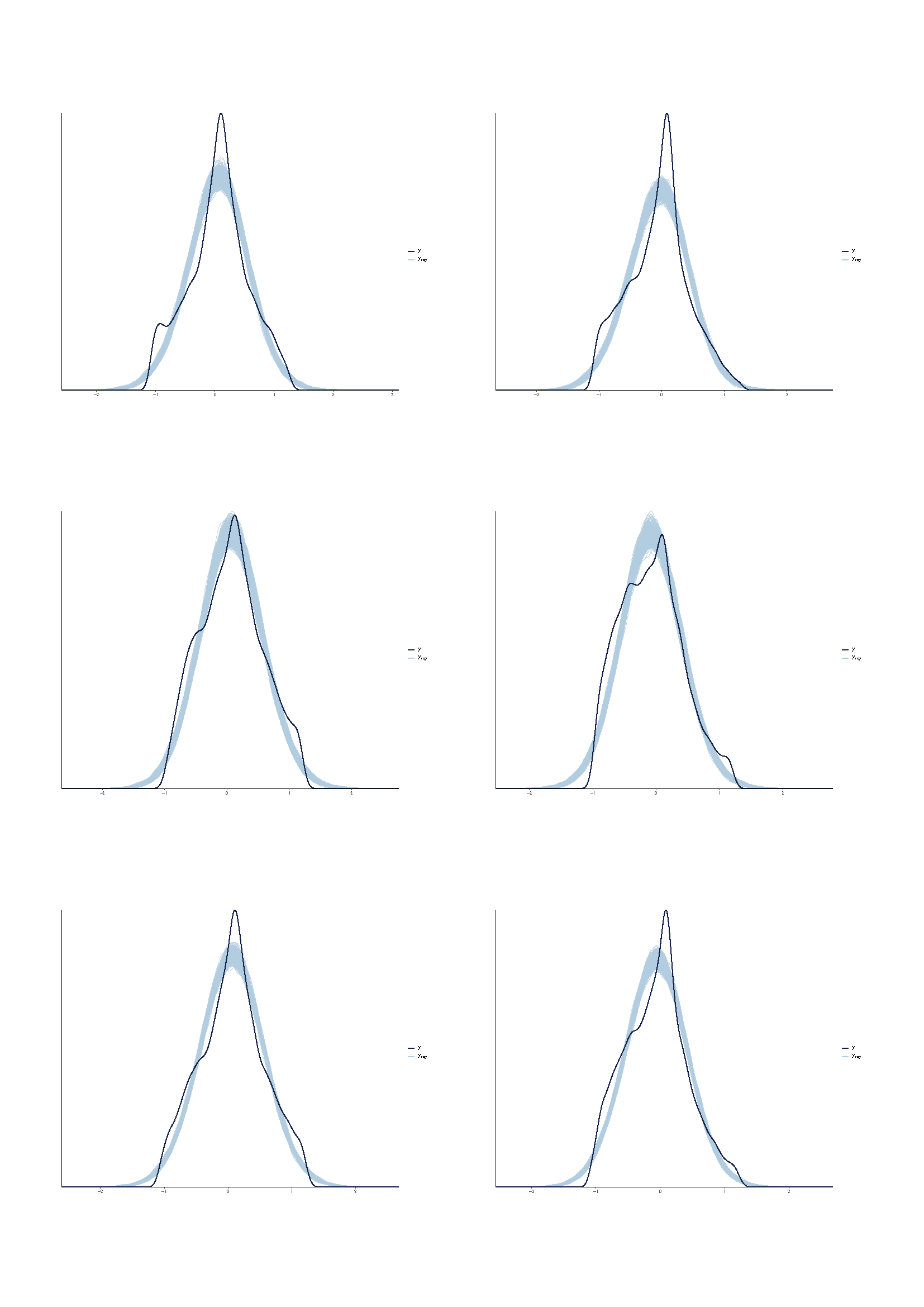

Supplement: S3 Fig — Consonance (left column) and Valence (right column) for Experiment 1 (top), Experiment 2 (middle) and Experiment 1&2 combined (bottom). The thin black line is the distribution of the observed outcomes and the blue lines represent the 1000 draws from the posterior predictive distribution. (TIFF) [file pone.0218570.s003.tiff]

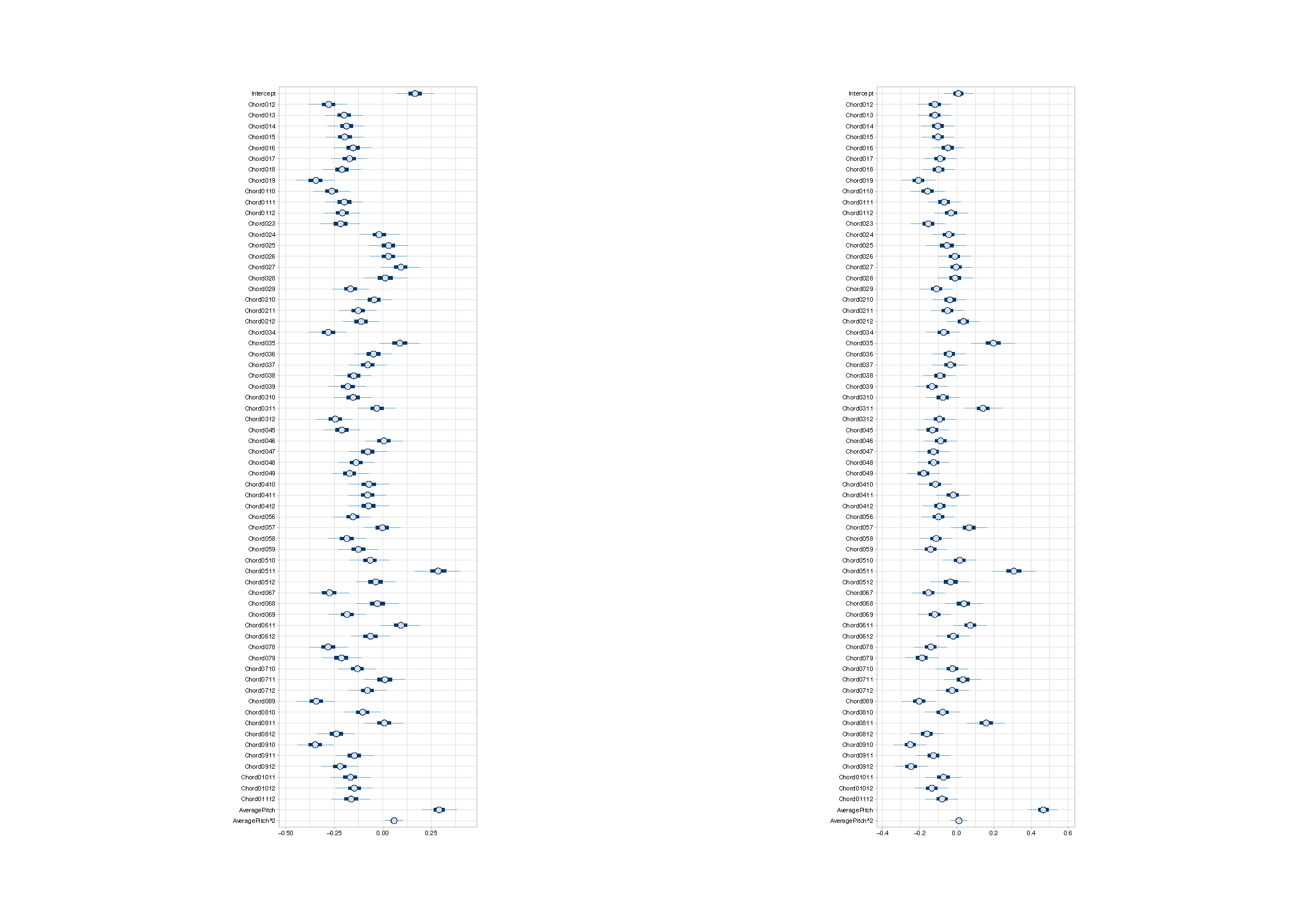

Supplement: S4 Fig — Mean consonance (left) and valence (right) ratings with 95% credibility intervals after controlling for a quadratic function of average pitch height. The thick darker blue line shows the 50% interval and the thinner light blue line shows the 95% interval. (TIFF) [file pone.0218570.s004.tiff]

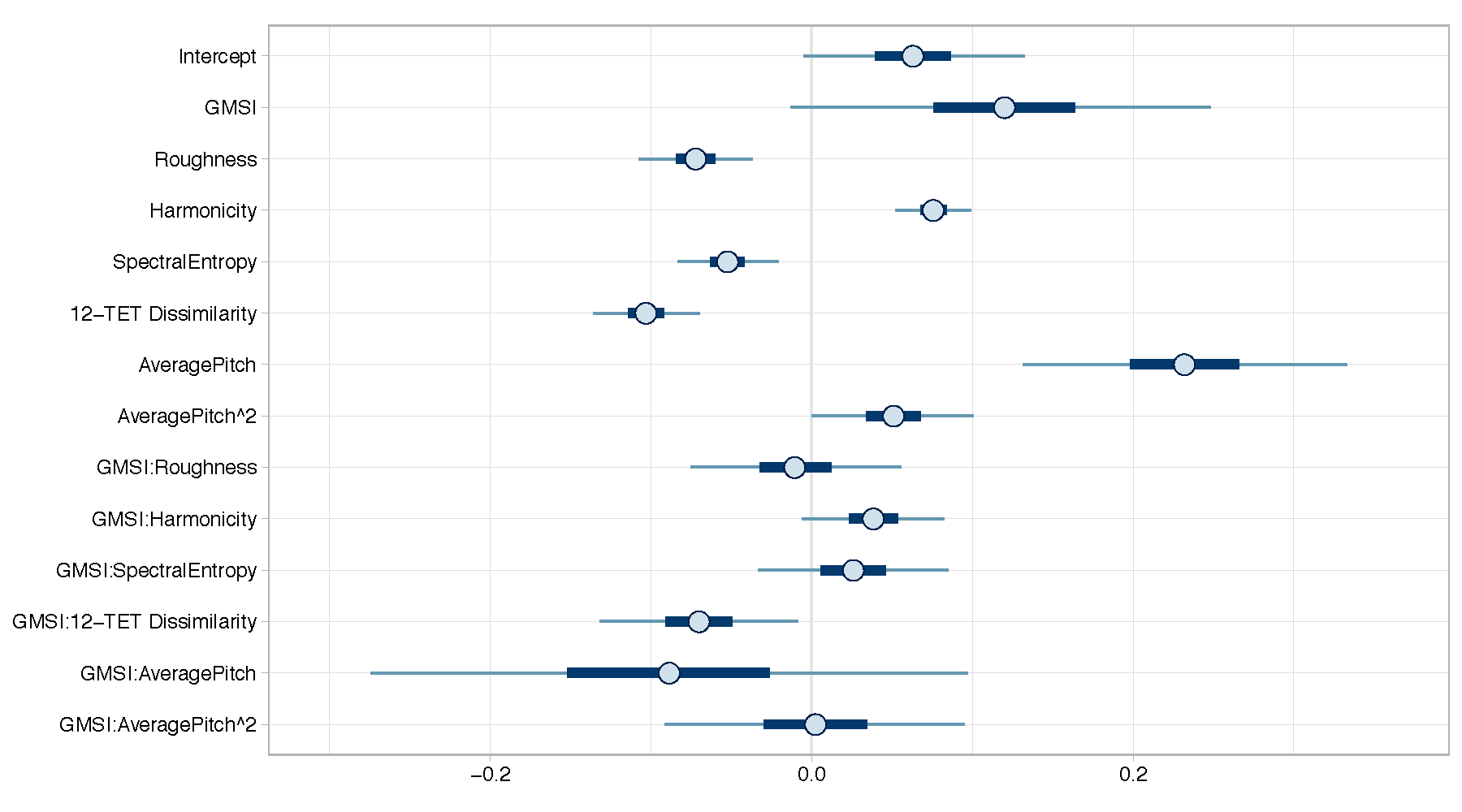

Supplement: S5 Fig — The thick darker blue line shows the 50% interval and the thinner light blue line shows the 95% interval. (TIFF) [file pone.0218570.s005.tiff]

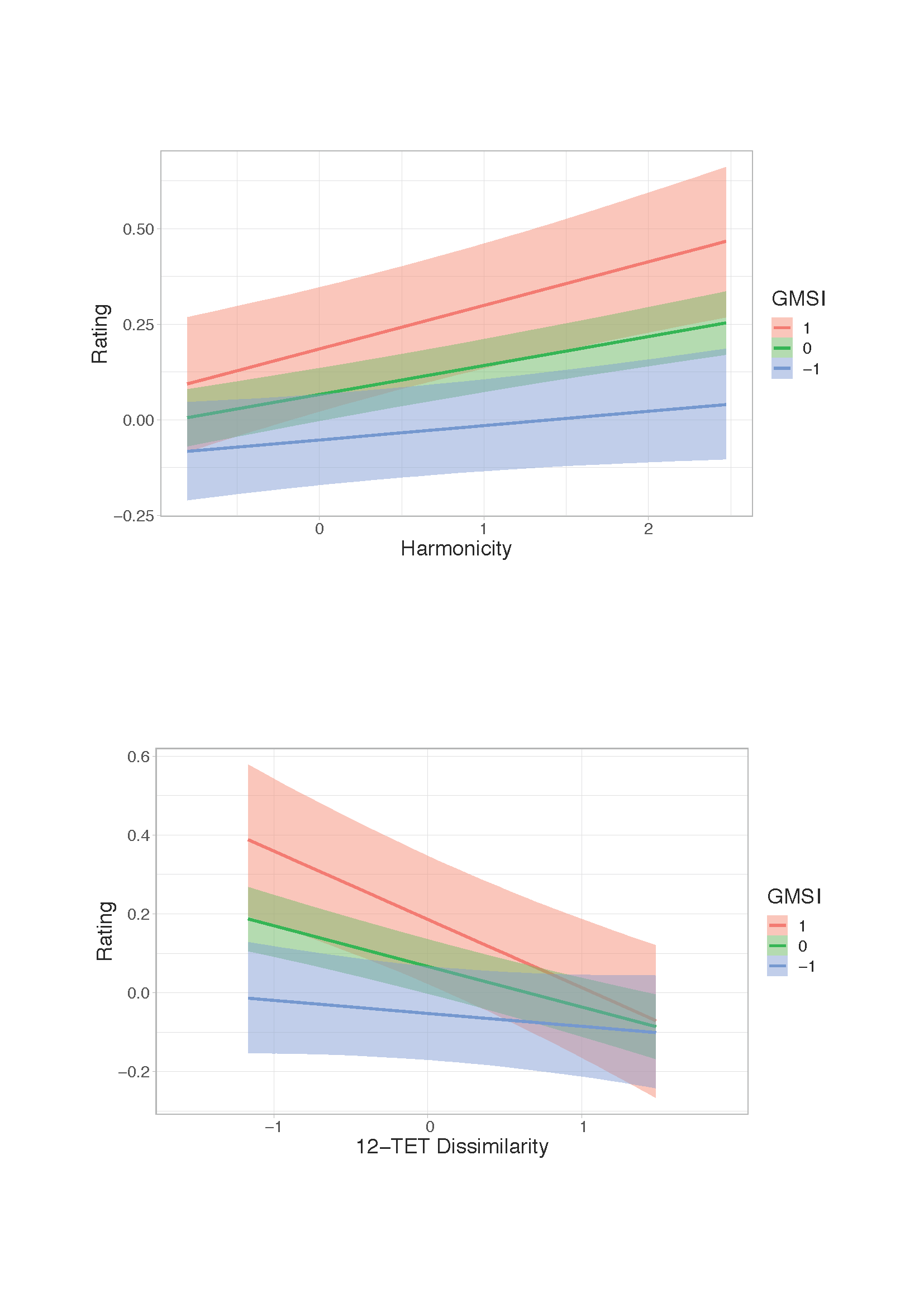

Supplement: S6 Fig — Levels of GMSI represent the mean (0), 1 SD above the mean (1) and 1 SD below the mean (-1). (TIFF) [file pone.0218570.s006.tiff]

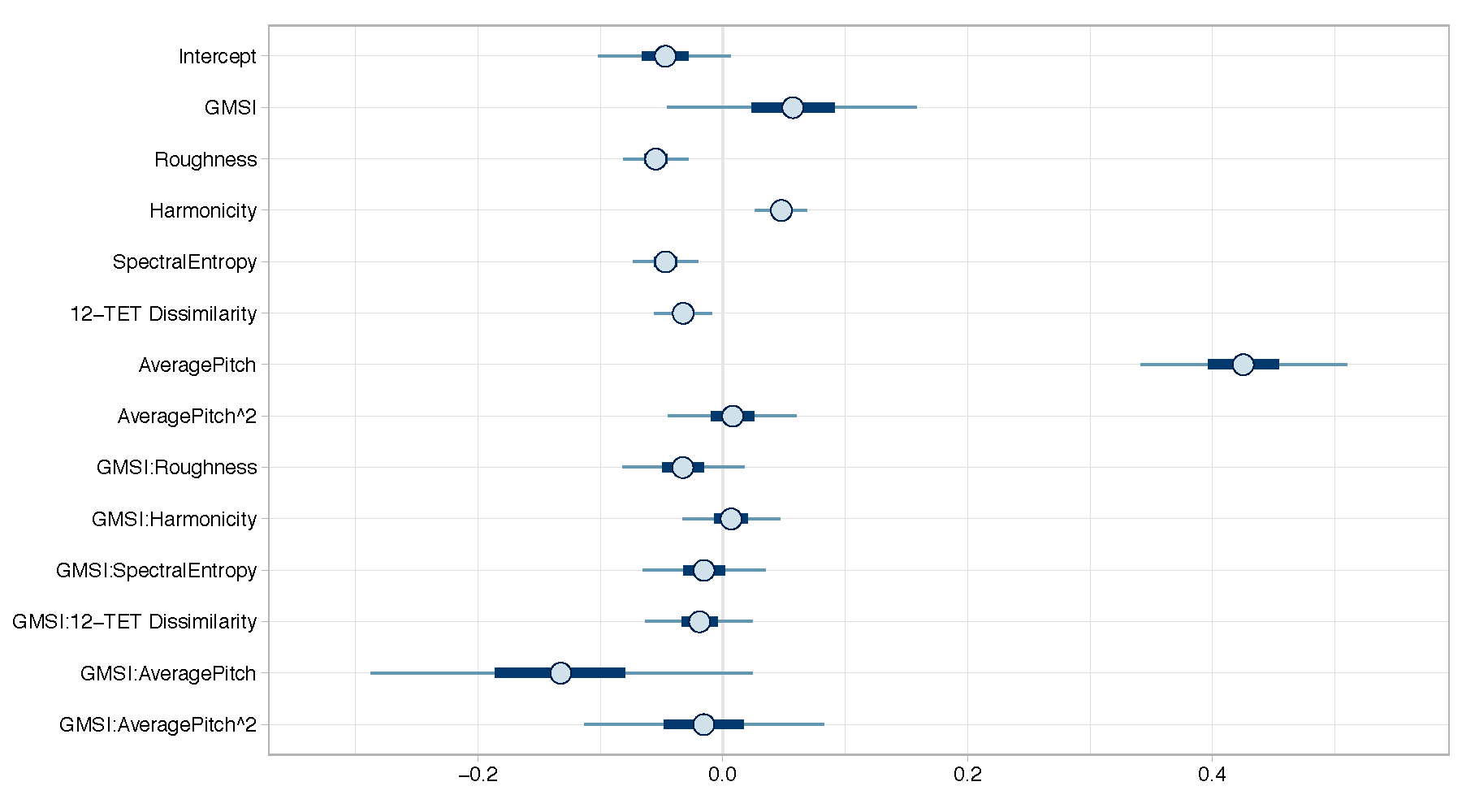

Supplement: S7 Fig — The thick darker blue line shows the 50% interval and the thinner light blue line shows the 95% interval. (TIFF) [file pone.0218570.s007.tiff]

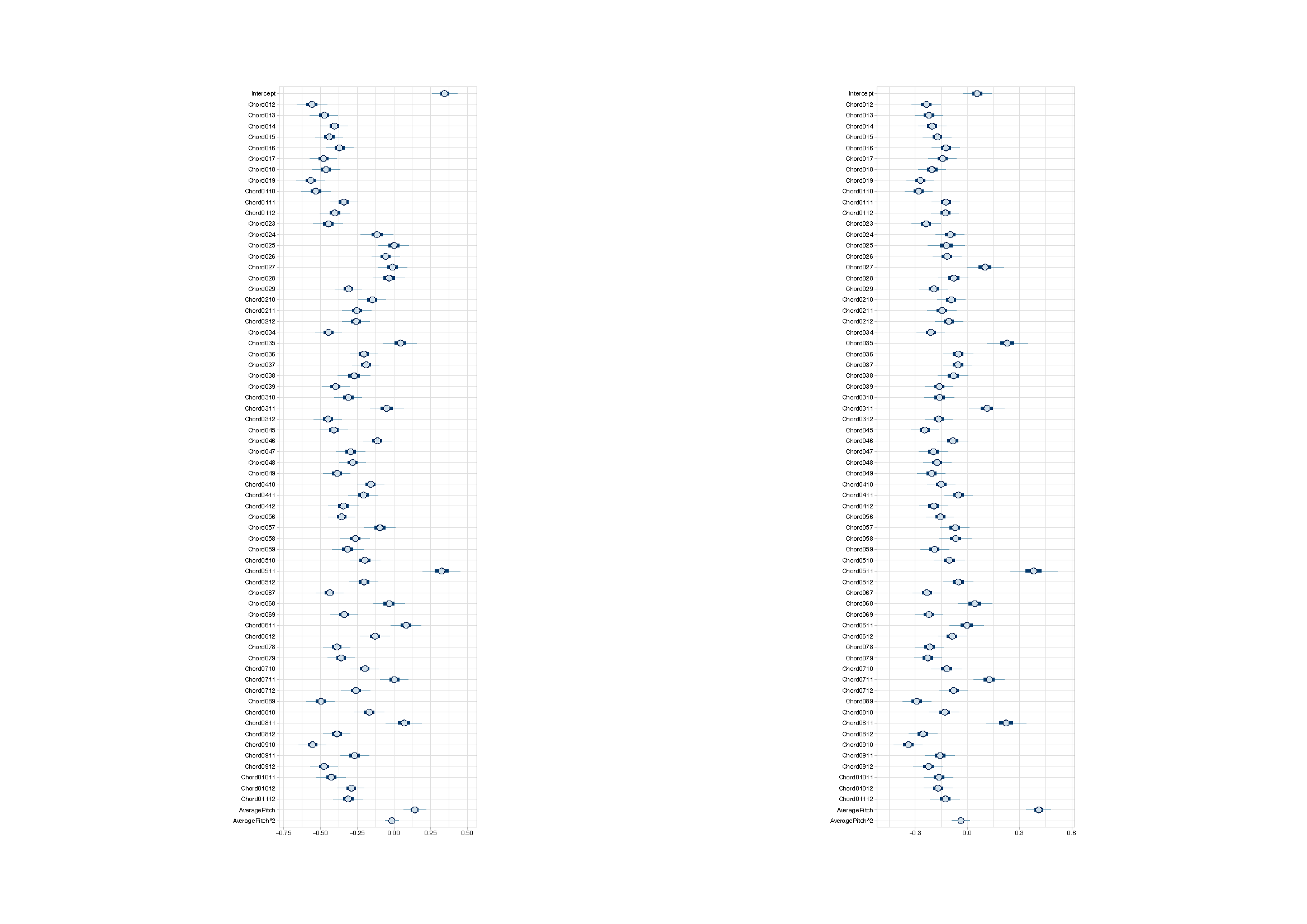

Supplement: S8 Fig — Mean consonance (left) and valence (right) ratings with 95% credibility intervals after controlling for a quadratic function of average pitch height. The thick darker blue line shows the 50% interval and the thinner light blue line shows the 95% interval. (TIFF) [file pone.0218570.s008.tiff]

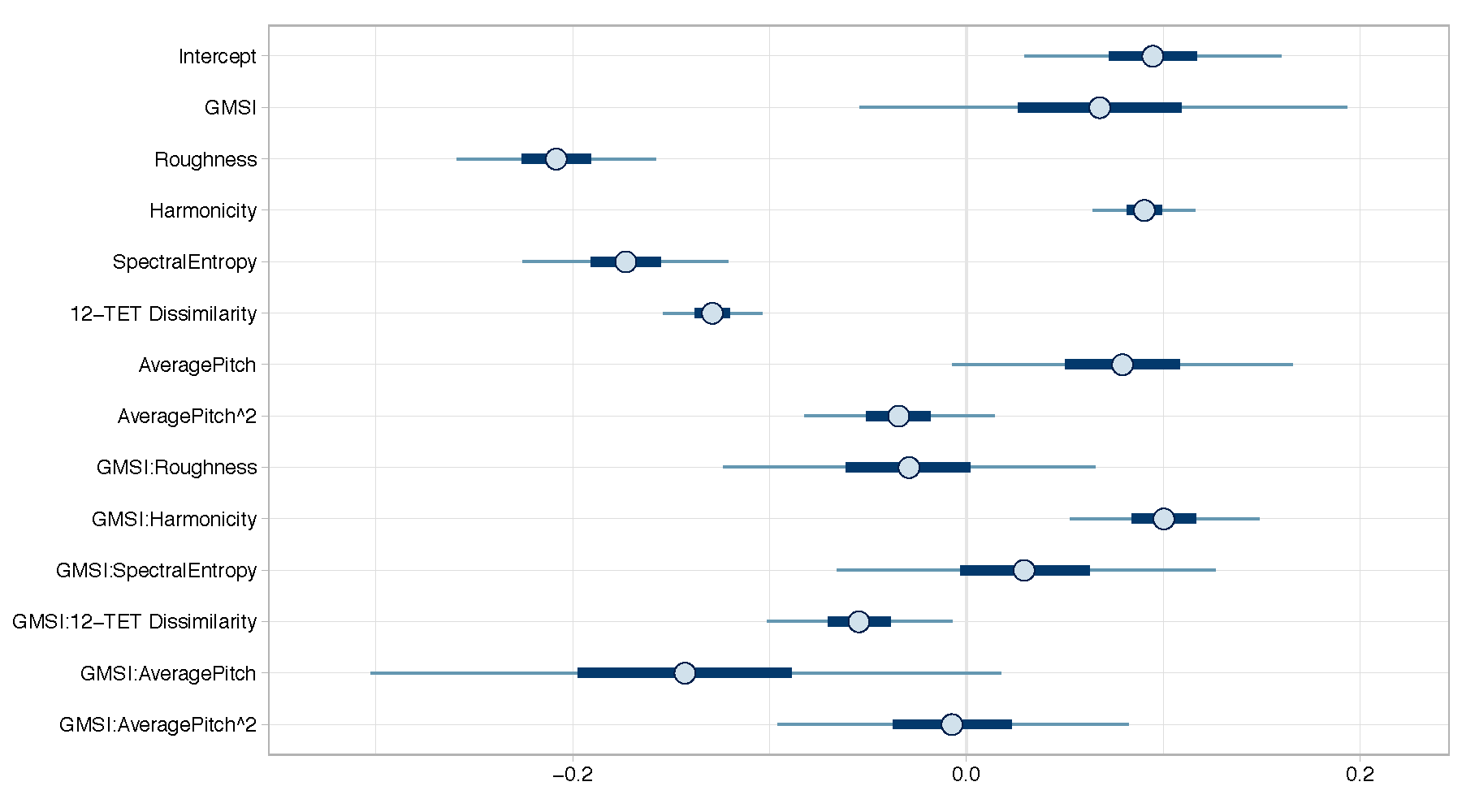

Supplement: S9 Fig — The thick darker blue line shows the 50% interval and the thinner light blue line shows the 95% interval. (TIFF) [file pone.0218570.s009.tiff]

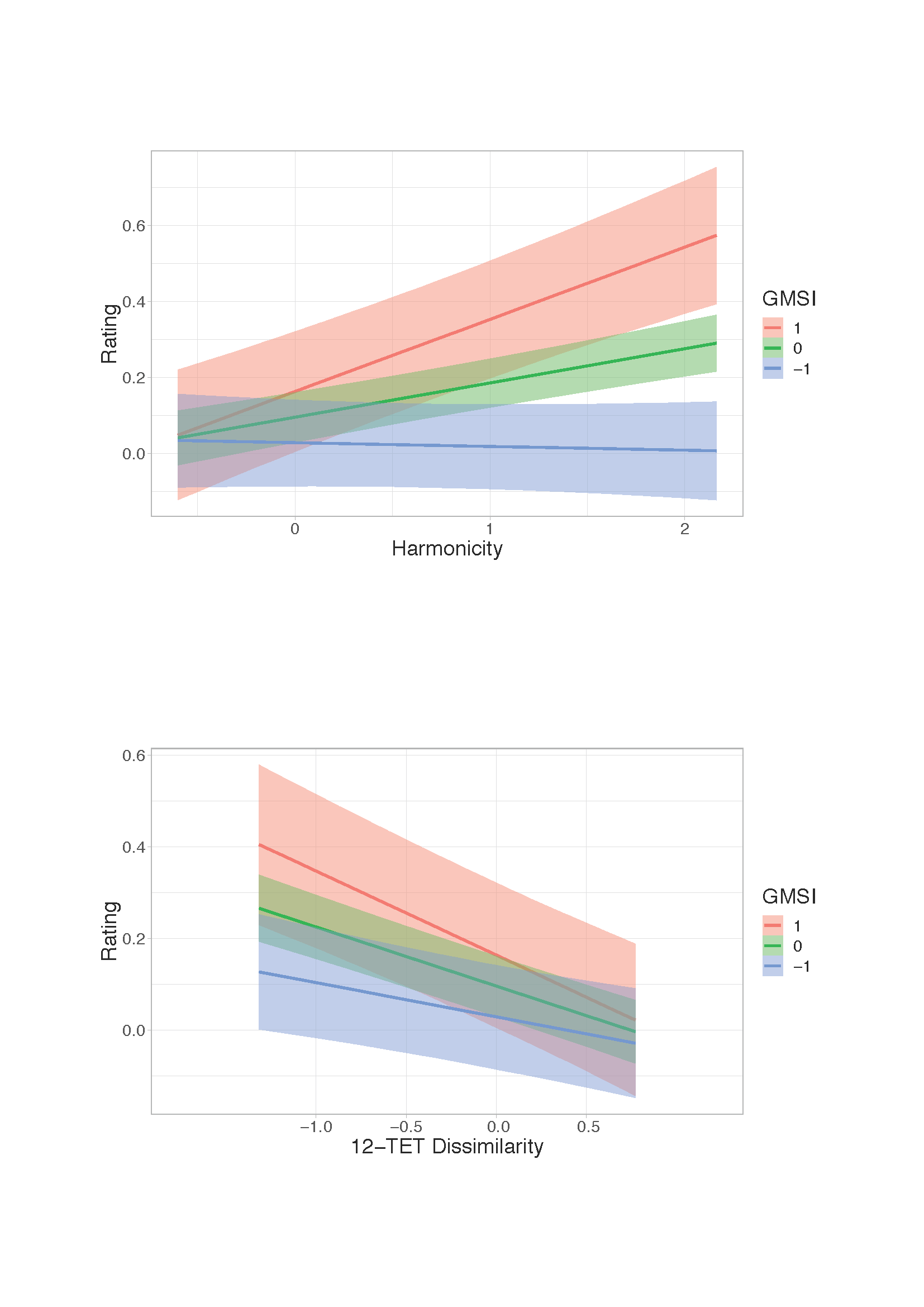

Supplement: S10 Fig — Levels of GMSI represent the mean (0), 1 SD above the mean (1) and 1 SD below the mean (-1). (TIFF) [file pone.0218570.s010.tiff]

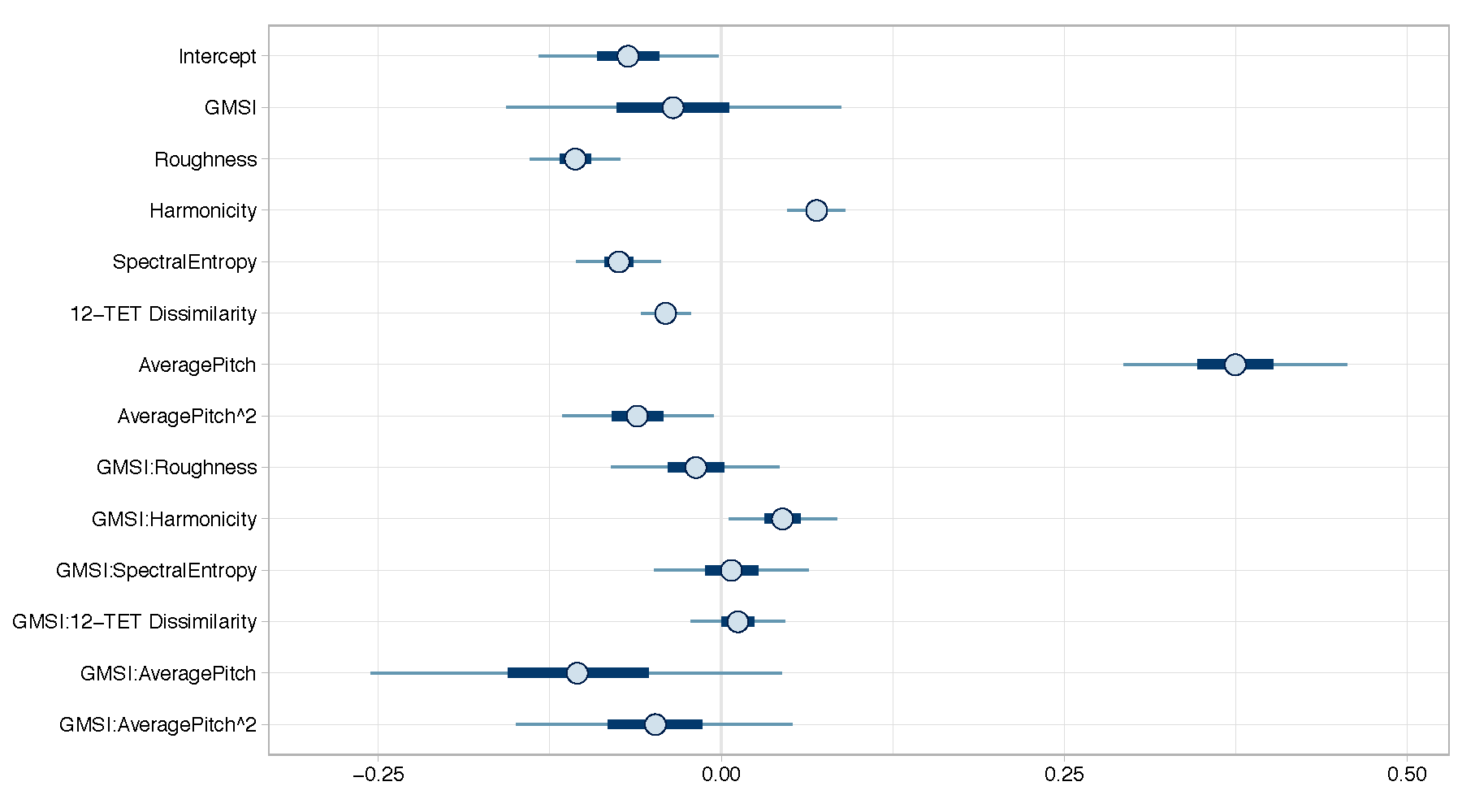

Supplement: S11 Fig — The thick darker blue line shows the 50% interval and the thinner light blue line shows the 95% interval. (TIFF) [file pone.0218570.s011.tiff]

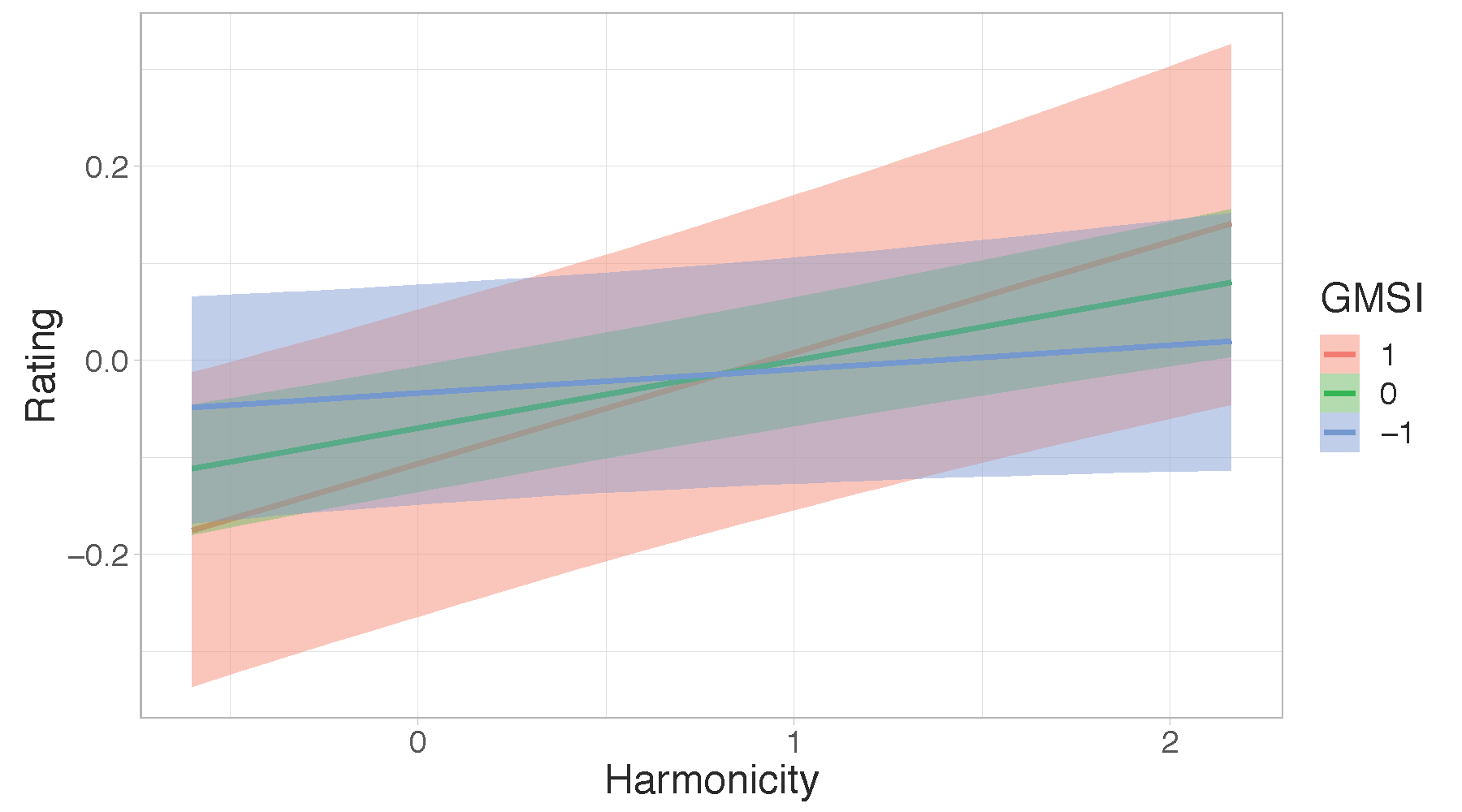

Supplement: S12 Fig — Levels of GMSI represent the mean (0), 1 SD above the mean (1) and 1 SD below the mean (-1). (TIFF) [file pone.0218570.s012.tiff]
