# Supplementary material for: Perception of affect in unfamiliar musical chords
Source: PLoS One. 2019 Jun 21;14(6):e0218570. doi: 10.1371/journal.pone.0218570 (PMC6588276; doi:10.1371/journal.pone.0218570)
Supplement: S1 File — (DOCX) [file pone.0218570.s013.docx]

# Supplementary materials

## Exploratory regression for effects of statistical learning

**S1_Table 1: Model output for Consonance.**

| Population-Level Effects | Estimate | Est. Error | CI Lower | CI Upper |
| --- | --- | --- | --- | --- |
| Intercept | 0.04 | 0.07 | -0.09 | 0.17 |
| ChordNo | -0.03 | 0.05 | -0.13 | 0.06 |
| ChordNo^2 | 0.02 | 0.01 | -0.00 | 0.03 |
| TrialNo | -0.04 | 0.02 | -0.07 | -0.01 |
| TrialNo^2 | -0.01 | 0.01 | -0.03 | 0.01 |

*Notes.* The expected value under the hypothesis lies outside the 95%-CI (credibility intervals). Estimate = mean. Estimate error = standard deviation of the posterior distribution. CI lower and CI upper are two-sided 95% credibility intervals.

**S1_Table 2: Model output for Valence.**

| Population-Level Effects | Estimate | Est. Error | CI Lower | CI Upper |
| --- | --- | --- | --- | --- |
| Intercept | -0.18 | 0.06 | -0.29 | -0.07 |
| ChordNo | 0.11 | 0.04 | 0.03 | 0.19 |
| ChordNo^2 | -0.02 | 0.01 | -0.04 | -0.01 |
| TrialNo | 0.02 | 0.02 | -0.01 | 0.05 |
| TrialNo^2 | 0.01 | 0.01 | -0.01 | 0.03 |

*Notes.* The expected value under the hypothesis lies outside the 95%-CI (credibility intervals). Estimate = mean. Estimate error = standard deviation of the posterior distribution. CI lower and CI upper are two-sided 95% credibility intervals.

## Marginal effect plots for average pitch height.

**S1_Fig 1: Consonance (left column) and Valence (right column) for Experiment 1 (top), Experiment 2 (middle) and Experiment 1&2 combined (bottom).**

## Histogram of mean ratings across all chords.

**S1_Fig 2: Consonance (left column) and Valence (right column) for Experiment 1 (top), Experiment 2 (middle) and Experiment 1&2 combined (bottom). The histograms show whether ratings are skewed or normal in their distribution.**

## Posterior distribution plots

**S1_Fig 3: Consonance (left column) and Valence (right column) for Experiment 1 (top), Experiment 2 (middle) and Experiment 1&2 combined (bottom). The thin black line is the distribution of the observed outcomes and the blue lines represent the 1000 draws from the posterior predictive distribution.**

# Model outcomes for Experiment 1 & 2

## Experiment 1

**S1_Fig 4: Mean consonance (left) and valence (right) ratings with 95% credibility intervals after controlling for a quadratic function of average pitch height. The thick darker blue line shows the 50% interval and the thinner light blue line shows the 95 % interval.**

**S1_Fig 5: Consonance model with all modelled parameters and 95% Bayesian credibility intervals for Experiment 1. The thick darker blue line shows the 50% interval and the thinner light blue line shows the 95 % interval.**

**S1_Table 3: Directed hypothesis testing for Consonance.**

| Hypothesis | Estimate | Est. Error | CI Lower | CI Upper | Evid. Ratio | Star |
| --- | --- | --- | --- | --- | --- | --- |
| Roughness < 0 | -0.07 | 0.02 | -Inf. | -0.04 | 35999 | * |
| Harmonicity > 0 | 0.08 | 0.01 | 0.06 | Inf. | Inf. | * |
| SpectralEntropy < 0 | -0.05 | 0.02 | -Inf. | -0.03 | 1027.57 | * |
| 12-TET Dissimilarity < 0 | -0.1 | 0.02 | -Inf. | -0.08 | Inf. | * |
| AveragePitch > 0 | 0.23 | 0.05 | 0.15 | Inf. | Inf. | * |
| AveragePitchE2 > 0 | 0.06 | 0.02 | 0.02 | Inf. | Inf. | * |
| GMSI:Roughness < 0 | -0.01 | 0.03 | -Inf. | 0.04 | 1.67 |  |
| GMSI:Harmonicity > 0 | 0.04 | 0.02 | 0.00 | Inf. | 21.78 | * |
| GMSI:SpectralEntropy < 0 | 0.03 | 0.03 | -0.02 | Inf. | 4.08 |  |
| GMSI:12-TET Dissimilarity > 0 | -0.07 | 0.03 | -Inf. | -0.02 | 74.47 | * |
| GMSI:AveragePitch < 0 | -0.09 | 0.09 | -Inf. | 0.07 | 4.83 |  |
| GMSI:AveragePitch^2 > 0 | 0.02 | 0.02 | 0.00 | Inf. | Inf. | * |

*Notes.* * =The expected value under the hypothesis lies outside the 95%-CI (credibility intervals). Estimate = mean. Estimate error = standard deviation of the posterior distribution. CI lower and CI upper are two-sided 95% credibility intervals. Evidence ratio = the posterior probability under the hypothesis against its alternative.

**S1_Fig 6: Marginal effects plots for GMSI and Harmonicity and GMSI and 12-TET Dissimilarity. Levels of GMSI represent the mean (0), 1 SD above the mean (1) and 1 SD below the mean (-1).**

**S1_Fig 7: Valence model with all modelled parameters and 95% Bayesian credibility intervals for Experiment 1. The thick darker blue line shows the 50% interval and the thinner light blue line shows the 95 % interval.**

**S1_Table 4: Directed hypothesis testing for Valence.**

| Hypothesis | Estimate | Est. Error | CI Lower | CI Upper | Evid. Ratio | Star |
| --- | --- | --- | --- | --- | --- | --- |
| Roughness < 0 | -0.05 | 0.01 | -Inf. | -0.03 | 35999 | * |
| Harmonicity > 0 | 0.05 | 0.01 | 0.03 | Inf. | Inf. | * |
| SpectralEntropy < 0 | -0.05 | 0.01 | -Inf. | -0.02 | 2570.43 | * |
| 12-TET Dissimilarity < 0 | -0.03 | 0.01 | -Inf. | -0.01 | 284.71 | * |
| AveragePitch > 0 | 0.43 | 0.04 | 0.36 | Inf. | Inf. | * |
| AveragePitchE2 > 0 | 0.18 | 0.04 | 0.13 | Inf. | Inf. | * |
| GMSI:Roughness < 0 | -0.03 | 0.03 | -Inf. | 0.01 | 9.04 |  |
| GMSI:Harmonicity > 0 | 0.01 | 0.02 | -0.03 | Inf. | 1.73 |  |
| GMSI:SpectralEntropy < 0 | -0.02 | 0.03 | -0.06 | Inf. | 0.37 |  |
| GMSI:12-TET Dissimilarity > 0 | -0.02 | 0.02 | -Inf. | 0.02 | 4.21 |  |
| GMSI:AveragePitch < 0 | -0.13 | 0.08 | -Inf. | 0.00 | 19.85 | * |
| GMSI:AveragePitch^2 > 0 | 0.02 | 0.02 | 0.00 | Inf. | Inf. | * |

*Notes.* * = The expected value under the hypothesis lies outside the 95%-CI (credibility intervals). Estimate = mean. Estimate error = standard deviation of the posterior distribution. CI lower and CI upper are two-sided 95% credibility intervals. Evidence ratio = the posterior probability under the hypothesis against its alternative.

## Experiment 2

**S1_Fig 8: Mean consonance (left) and valence (right) ratings with 95% credibility intervals after controlling for a quadratic function of average pitch height. The thick darker blue line shows the 50% interval and the thinner light blue line shows the 95 % interval.**

**S1_Fig 9: Consonance model with all modelled parameters and 95% Bayesian credibility intervals for Experiment 2. The thick darker blue line shows the 50% interval and the thinner light blue line shows the 95 % interval.**

**S1_Table 5: Directed hypothesis testing for Consonance.**

| Hypothesis | Estimate | Est. Error | CI Lower | CI Upper | Evid. Ratio | Star |
| --- | --- | --- | --- | --- | --- | --- |
| Roughness < 0 | -0.21 | 0.03 | -Inf. | -0.17 | Inf. | * |
| Harmonicity > 0 | 0.09 | 0.01 | 0.07 | Inf. | Inf. | * |
| SpectralEntropy < 0 | -0.17 | 0.03 | -Inf. | -0.13 | Inf. | * |
| 12-TET Dissimilarity < 0 | -0.13 | 0.01 | -Inf. | -0.13 | Inf. | * |
| AveragePitch > 0 | 0.08 | 0.04 | 0.01 | Inf. | 27.41 | * |
| AveragePitchE2 > 0 | 0.01 | 0.01 | 0.00 | Inf. | Inf. | * |
| GMSI:Roughness < 0 | -0.03 | 0.05 | -Inf. | 0.05 | 2.78 |  |
| GMSI:Harmonicity > 0 | 0.1 | 0.02 | 0.06 | Inf. | 11999 | * |
| GMSI:SpectralEntropy < 0 | 0.03 | 0.05 | 0.05 | Inf. | 2.69 |  |
| GMSI:12-TET Dissimilarity > 0 | -0.05 | 0.02 | -Inf. | -0.02 | 80.08 | * |
| GMSI:AveragePitch < 0 | -0.14 | 0.08 | -Inf. | -0.01 | 24.00 | * |
| GMSI:AveragePitch^2 > 0 | 0.03 | 0.03 | 0.00 | Inf. | Inf. | * |

*Notes.* The expected value under the hypothesis lies outside the 95%-CI (credibility intervals). Estimate = mean. Estimate error = standard deviation of the posterior distribution. CI lower and CI upper are two-sided 95% credibility intervals. Evidence ratio = the posterior probability under the hypothesis against its alternative.

**S1_Fig 10: Marginal effects plots for GMSI and Harmonicity and GMSI and 12-TET Dissimilarity. Levels of GMSI represent the mean (0), 1 SD above the mean (1) and 1 SD below the mean (-1).**

**S1_Fig 11: Valence** **model with all modelled parameters and 95% Bayesian credibility intervals for Experiment 2. The thick darker blue line shows the 50% interval and the thinner light blue line shows the 95 % interval.**

**S1_Table 6: Directed hypothesis testing for Valence.**

| Hypothesis | Estimate | Est. Error | CI Lower | CI Upper | Evid. Ratio | Star |
| --- | --- | --- | --- | --- | --- | --- |
| Roughness < 0 | -0.11 | 0.02 | -Inf. | -0.08 | Inf. | * |
| Harmonicity > 0 | 0.07 | 0.01 | 0.05 | Inf. | Inf. | * |
| SpectralEntropy < 0 | -0.07 | 0.02 | -Inf. | -0.05 | Inf. | * |
| 12-TET Dissimilarity < 0 | -0.04 | 0.01 | -Inf. | -0.03 | Inf. | * |
| AveragePitch > 0 | 0.37 | 0.04 | 0.31 | Inf. | Inf. | * |
| AveragePitchE2 > 0 | 0.14 | 0.03 | 0.09 | Inf. | Inf. | * |
| GMSI:Roughness < 0 | -0.02 | 0.03 | -Inf. | 0.03 | 2.68 |  |
| GMSI:Harmonicity > 0 | 0.04 | 0.02 | 0.01 | Inf. | 74.79 | * |
| GMSI:SpectralEntropy < 0 | -0.01 | 0.03 | -0.04 | Inf. | 1.52 |  |
| GMSI:12-TET Dissimilarity > 0 | 0.01 | 0.02 | -Inf. | 0.04 | 0.32 |  |
| GMSI:AveragePitch < 0 | -0.1 | 0.08 | -Inf. | 0.02 | 10.85 |  |
| GMSI:AveragePitch^2 > 0 | 0.02 | 0.02 | 0.00 | Inf. | Inf. | * |

*Notes.* * = The expected value under the hypothesis lies outside the 95%-CI (credibility intervals). Estimate = mean. Estimate error = standard deviation of the posterior distribution. CI lower and CI upper are two-sided 95% credibility intervals. Evidence ratio = the posterior probability under the hypothesis against its alternative.

**S1_Fig 12: Marginal effects plot for GMSI and Harmonicity. Levels of GMSI represent the mean (0), 1 SD above the mean (1) and 1 SD below the mean (-1).**
